# Decoding inflammation, its causes, genomic responses, and emerging countermeasures

**DOI:** 10.1111/sji.12812

**Published:** 2019-08-28

**Authors:** Jacek Hawiger, Jozef Zienkiewicz

**Affiliations:** ^1^ Immunotherapy Program at Vanderbilt University School of Medicine Nashville TN USA; ^2^ Division of Allergy, Pulmonary and Critical Care Medicine, Department of Medicine Vanderbilt University School of Medicine Nashville TN USA; ^3^ Department of Veterans Affairs Tennessee Valley Health Care System Nashville TN USA; ^4^ Department of Molecular Physiology and Biophysics Vanderbilt University School of Medicine Nashville TN USA

**Keywords:** allergy, atherosclerosis, autoimmunity, diabetes, genome, hepatitis, lung inflammation, metabolic syndrome, nuclear transport, transcription, trauma

## Abstract

Inflammation is the mechanism of diseases caused by microbial, autoimmune, allergic, metabolic and physical insults that produce distinct types of inflammatory responses. This aetiologic view of inflammation informs its classification based on a cause‐dependent mechanism as well as a cause‐directed therapy and prevention. The genomic era ushered in a new understanding of inflammation by highlighting the cell's nucleus as the centre of the inflammatory response. Exogenous or endogenous inflammatory insults evoke genomic responses in immune and non‐immune cells. These genomic responses depend on transcription factors, which switch on and off a myriad of inflammatory genes through their regulatory networks. We discuss the transcriptional paradigm of inflammation based on denying transcription factors’ access to the nucleus. We present two approaches that control proinflammatory signalling to the nucleus. The first approach constitutes a novel intracellular protein therapy with bioengineered physiologic suppressors of cytokine signalling. The second approach entails control of proinflammatory transcriptional cascades by targeting nuclear transport with a cell‐penetrating peptide that inhibits the expression of 23 out of the 26 mediators of inflammation along with the nine genes required for metabolic responses. We compare these emerging anti‐inflammatory countermeasures to current therapies. The transcriptional paradigm of inflammation offers nucleocentric strategies for microbial, autoimmune, metabolic, physical and other types of inflammation afflicting millions of people worldwide.

Do not go where the path may lead, go instead where there is no path and leave a trail. Ralph Waldo Emerson (1803‐1882)

## 1 INTRODUCTION

**Figure 1:**
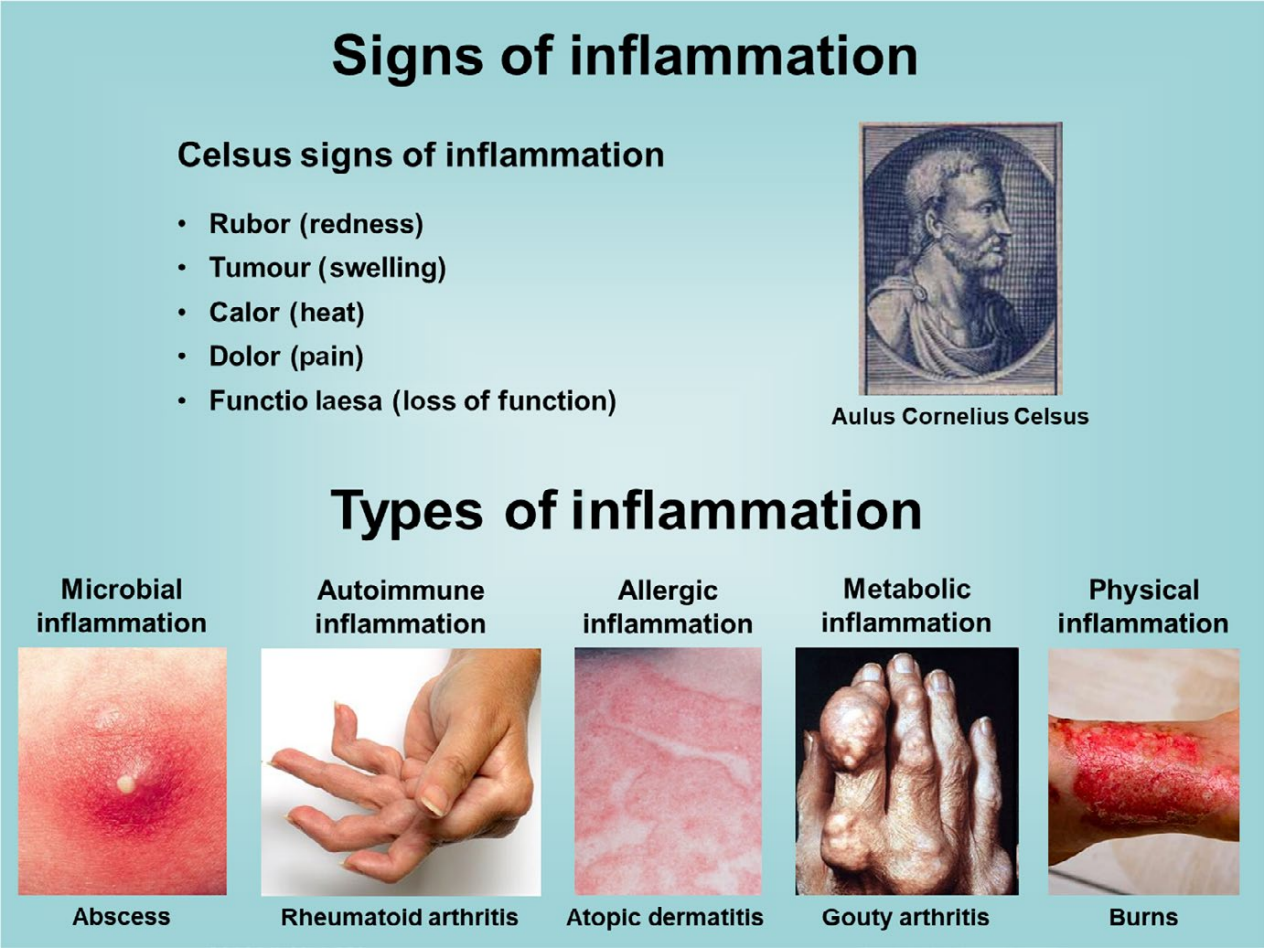
Signs and types of inflammation. Photographs obtained by courtesy of: Bibliotheca Augustana, Augsburg, Germany (Celsus copperplate); MidlevelU (Abscess); MIMS (Rheumatoid Arthritis); Royal Sea (Atopic Dermatitis); Johns Hopkins Arthritis Center (Gouty Arthritis); and Healthline (Burns)

Inflammation is the mechanism of human diseases displaying the five classic inflammatory signs: redness, swelling, heat, pain and subsequent loss of organ function (Figure 1). Aulus Cornelius Celsus and his followers already described these signs in the 1st and 2nd century AD. The underlying mechanisms of inflammation remained unknown until the 19th century when Ilya I. Mechnikov discovered phagocytes, and subsequently defined the inflammatory state as a “lesion of the vessels which are attacked by the irritating cause.”^1^ Depending on the nature of the “irritating cause,” we distinguish the following types of inflammation: microbial, autoimmune, allergic, metabolic and physical inflammation displayed in Figure 1. *Microbial inflammation*, caused by bacteria, viruses, fungi and protozoa, mediates abscess, pneumonia, sepsis, Ebola Haemorrhagic Fever, human immunodeficiency virus infection and other infectious diseases. *Autoimmune inflammation*, caused by an aberrant autoimmune attack by autoantibodies or autoreactive B and T cells, mediates rheumatoid arthritis, Type 1 diabetes, multiple sclerosis, Crohn's disease, psoriasis, systemic lupus erythematosus and other autoimmune diseases. *Allergic inflammation*, caused by allergens, mediates atopic dermatitis, allergic rhinitis, asthma and other allergic diseases. *Metabolic inflammation*, caused by overfeeding and excessive accumulation of metabolites (eg uric acid or cholesteryl esters) resulting from inborn or acquired metabolic dysfunction, mediates gout or atherosclerosis, among other metabolic diseases. The fifth type, *physical inflammation*, is caused by trauma, burns and radiation (Table 1).

**Table 1:** The cause‐based classification of inflammation

Type of inflammation	Cause of inflammation	Examples of diseases mediated by given type of inflammation
Microbial inflammation	Bacteria, Fungi, Viruses, and Protozoa	Abscess; Pneumonia; Sepsis; Ebola Haemorrhagic Fever
Autoimmune inflammation	Aberrant Autoimmune Attack by Autoantibodies and/or Autoreactive B and T Cells	Type 1 Diabetes; Multiple Sclerosis; Rheumatoid Arthritis; Psoriasis; Systemic Lupus Erythematosus
Allergic inflammation	Allergens (eg pollen, dust mites, animal dander, fungi, insects’ bites and stings)	Atopic Dermatitis/Eczema; Hay Fever; Asthma; Contact Dermatitis; Anaphylaxis; Drug Hypersensitivity Reactions
Metabolic inflammation	Excessive Accumulation of Metabolites (eg cholesteryl esters or uric acid)	Atherosclerosis; Gout; Phenylketonuria
Physical inflammation	Trauma, Burns or Radiation	Post‐traumatic Injury; Chemical, Electric and Thermal (scalding) Burns; Radiation Injury
Constitutive inflammation	Inborn Errors of Innate Immunity	Autoinflammatory diseases such as Familial Mediterranean Fever; Aicardi‐Goutieres Syndrome; NEMO Mutation‐Linked Autoinflammatory Intestinal and Skin Disease

The sixth type of inflammation, which we term *constitutive inflammation* (not displayed in Figure 1 but listed in Table 1), is caused by the inborn errors of innate immunity that underlie autoinflammatory diseases. These diseases display the overt signs of inflammation without either an apparent source of infection or the markers of the autoimmune process. The autoinflammatory diseases include the following: (a) Familial Mediterranean Fever (FMF) and other cryopyrin‐associated periodic syndromes (CAPS),^2^ (b) Aicardi‐Goutières syndrome and other Type 1 interferonopathies caused by gain‐of‐function mutations of the intracellular nucleic acid sensors resulting in constitutively active Type 1 interferon signalling,^3^ (c) inflammatory disorder of the skin and bones caused by loss‐of‐function mutations of the interleukin (IL)‐1 receptor antagonist^4^, ^5^ and (d) intestinal and skin inflammatory disorders caused by the deletion mutation of the carboxy‐terminal segment of the NF‐κB essential modulator (NEMO) responsible for loss of control by the physiologic suppressor, ubiquitin modifier A20. It results in constitutively active NF‐κB signalling.^6^ Thus, the aetiologic view of the six types of inflammation informs not only its cause‐dependent mechanism but also a cause‐directed therapy. The alternate term “sterile inflammation” denotes an inflammation distinct from that caused by microbial agents, thereby accounting for other types of inflammation listed in Table 1.^7^, ^8^

This review focuses on signalling to the cell's nucleus in response to the different causes of inflammation. Proinflammatory signalling is transmitted by the distinct transcriptional cascades that activate hundreds of genes encoding mediators and suppressors of inflammation. We analyse the transcriptional paradigm of inflammation based on transcription factors’ denial of access to the inflammatory regulome in the nucleus of immune and non‐immune cells participating in the inflammatory response. Finally, we compare the classic and emerging anti‐inflammatory countermeasures tested in microbial, autoimmune, metabolic and physical inflammation.

## 2 DETECTORS AND MEDIATORS OF INFLAMMATORY INSULTS

Non‐immune cells, such as skin keratinocytes, mucosal epithelial cells and vascular endothelial cells, serve as an organ‐specific “barrier” while being the first‐line sentinels for the exogenous and endogenous causes of inflammation.^9^ Together with polymorphonuclear leucocytes (neutrophils, eosinophils, basophils) and the strategically positioned macrophages, dendritic cells, Natural Killer (NK) cells and groups 1, 2 and 3 innate lymphoid cells (ILC), and non‐immune cells alert the immune system to the presence of inflammation‐causing irritants and modulate the inflammatory response.^10^, ^11^, ^12^, ^13^, ^14^, ^15^, ^16^, ^17^ These innate immunity effectors establish a tight communication with B and T cells constituting adaptive immunity. The effectors provide the signalling relays in inflammation caused by allergic, autoimmune and microbial insults. For example, in allergic inflammation, epithelial cells “irritated” by allergens produce interleukin (IL)‐33 that activates lung ILC2. In turn, these ILC produce type 2 interleukins, IL‐5 and IL‐13, which initiate an adaptive Th2 response.^18^ In microbial inflammation caused by staphylococci that produce immunotoxins, termed superantigens, a tight signalling synapse is formed between the antigen‐presenting major histocompatibility complex class II expressed on dendritic cells or macrophages, and the T cell receptor Vβ.3, 12, 14 and 17 in human CD4 T cells.^19^ The staphylococcal superantigen‐induced synapse is responsible for the robust production of IL‐2, IL‐4, interferon γ (IFNγ) and tumour necrosis factor (TNF)‐α, among other blood cytokines and chemokines. IL‐2 and other cytokines destabilize microvascular endothelial cells.^20^ These cells’ dysfunction and injury lead to systemic inflammation known as toxic shock syndrome that can be controlled by novel intracellular protein and peptide therapies^21^, ^22^ (see below). In autoimmune inflammation, B cells expressing the transcription factor T‐bet, also known as age‐associated B cells (ABC), participate in response to the ligands for Toll‐like receptors 7 and 9, and cytokines by producing in mice IgG2_a/c_ directed against intracellular viral pathogens.^23^ The T‐bet^+^ memory B cells persist in the spleen and are expanded in patients with autoimmune diseases.

As the body's response to harmful causes, inflammation is initially beneficial by: (a) mobilizing the innate and adaptive immune systems, (b) assisting the body in containing the cause of inflammation and (c) healing damaged organs. This “physiologic” side of inflammation^24^ depends on the availability of endogenous suppressors of proinflammatory signalling pathways, as we will discuss below. However, when physiologic suppressors fail, uncontrolled inflammation can acutely or chronically lead to apoptosis, necrosis, fibrosis and, ultimately, end‐stage organ destruction.^25^

Innate and adaptive immune cells respond to proinflammatory insults by producing intracellular and extracellular inflammatory mediators while also displaying their cognate receptors. Therefore, the inflammatory response is perpetuated by autoregulatory feed‐forward loops. Mediators include cytokines, chemokines, haemopoietic/vascular growth factors and cognate receptors. Moreover, intercellular and intracellular inflammatory responses are mediated by cell adhesion molecules, such as vascular cell adhesion molecule‐1, integrin α4, complement proteins and signal transducers, for example COX‐2.^26^, ^27^, ^28^, ^29^, ^30^, ^31^ The genes that encode all these mediators are regulated by transcription factors. The activation of proinflammatory transcription factors is controlled by physiologic suppressors of cytokine signalling (SOCS) and other intracellular adaptors (eg ubiquitin‐modifying dual enzyme termed A20 protein). However, the capacity of these physiologic brakes is time‐limited, thereby allowing the sufficient transit of activated transcription factors to the nucleus. This transit is carried out by the nuclear transport shuttle proteins termed importins/karyopherins. These proteins are essential for proinflammatory transcription factors’ access to the genome.

## 3 THE INFLAMMATORY REGULATORY NETWORK (“REGULOME”) IN THE NUCLEUS—THE COMMAND CENTRE OF INFLAMMATION

The cell's nucleus is the receiver, processor and dispatcher of signals evoking, maintaining and extinguishing inflammation. The human genome contains approximately 23 000 genes, among them 19 000 encoding proteins. These genes comprise about 1%‐2% of the total human DNA sequence, wherein protein‐coding genes are interspersed among the estimated 500 000 regulatory elements (“regulome”). Many of these regulatory elements are strategically positioned within DNA's 10 000 loops. The regulome interacts with approximately 1850 transcription factors belonging to 39 families.^32^, ^33^, ^34^, ^35^, ^36^ Transcription factors regulate the genes through binding to their promoters, enhancers and super enhancers, thereby demarcating the cohorts of cell‐identity genes in each cell.^37^, ^38^, ^39^, ^40^ Clearly, transcription factors are super‐regulators mobilized to initiate a profound reprogramming of the human genome in response to proinflammatory insults. Transcription factors also regulate the expression and action of long non‐coding RNAs and microRNA,^41^, ^42^ thereby increasing the inflammatory regulome's complexity. Since the mid‐1990s, we focused our studies on controlling the nuclear transport of proinflammatory stress‐responsive and metabolic transcription factors (see below).

## 4 TRANSCRIPTIONAL PARADIGM OF INFLAMMATION

Stopping transcription factors on their way to the nucleus offers a simple approach to establish their role in inflammation. Our initial evidence indicated that the expression of the *Il2* gene in human T cells can be controlled at the nuclear import level. The inhibition of nuclear translocation through the competitive recognition of the peptide mimicking nuclear localization sequence (NLS) of at least three transcription factors (NF‐κB, cFos and NFAT) suppressed the expression of the gene encoding IL‐2, the key immunoregulatory cytokine for B and T cells.^43^, ^44^ Based on these findings, we proposed the transcriptional paradigm of inflammation.^45^ It was supported by the in vivo suppression of the *Tnfα* gene regulated by the NF‐κB, NFAT and NTF2/Jun, and the *Ifnγ* gene controlled by NF‐κB, NFAT and STAT1 in a murine model of lethal endotoxic shock caused by lipopolysaccharide (LPS). LPS is one of the most potent inducers of microbial inflammation (see below). Strikingly, impeding these transcription factors’ access to the genome was accompanied by a 90% gain in survival.^46^

Later, the consortium of investigators, studying the host's response to injury, demonstrated that, in healthy human volunteers, a single low dose (5 ng/kg) of parenterally administered LPS, a Toll‐like receptor 4 (TLR4) ligand,^47^, ^48^ evoked a twofold increase in the expression of 4533 genes in circulating leucocytes, a transcriptional response termed a “genomic storm.”^49^ At the same time, LPS repressed a significant number of genes, indicating the state of global genomic reprogramming. It is now understood that the regulatory network of inflammatory transcription factors assembles in the cell's nucleus in order to govern the expression of hundreds of genes in response to proinflammatory insults.^50^

## 5 PROINFLAMMATORY TRANSCRIPTIONAL CASCADES CARRY SIGNALS TO THE NUCLEUS IN RESPONSE TO THE “IRRITATING CAUSES”

**Figure 2:**
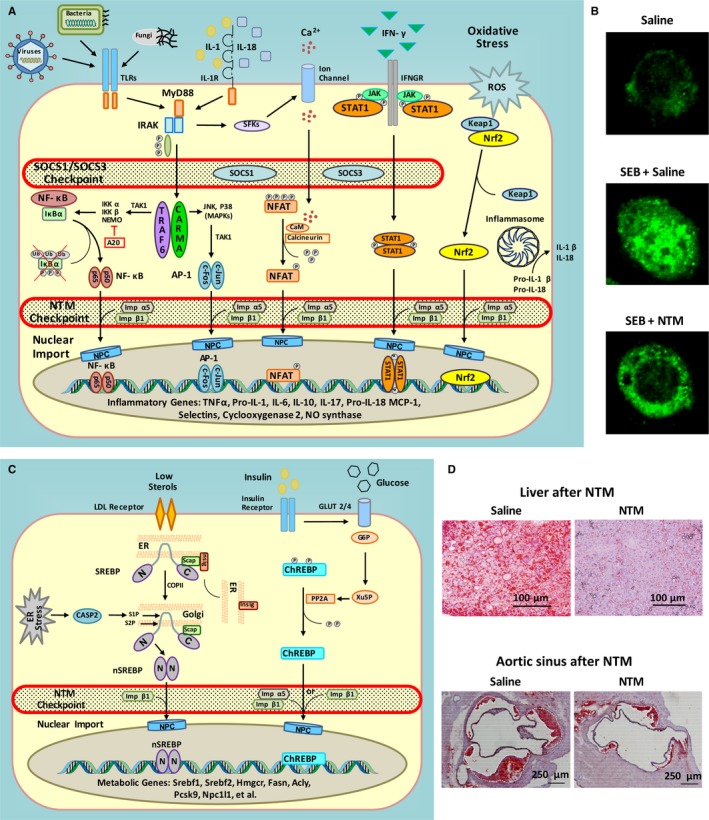
Inflammatory transcriptional cascades with checkpoints controlled by Suppressors of Cytokine Signalling (SOCS) and NTMs. A, Five signalling cascades mediated by proinflammatory stress‐responsive transcription factors and Nrf2 converge in nuclear pore complex (NPC). B, Example of nuclear translocation of NF‐κB Rel A (green fluorescence) in lymphocytes collected from bronchoalveolar lavage of mice challenged with superantigen, staphylococcal enterotoxin B (SEB) and treated with Nuclear Transport Modifier (NTM, cSN50 peptide). Please note the paucity of NF‐κB Rel A in the cell's nucleus of NTM‐treated animal (adapted from Liu *et al*, Mol. Ther., 2009). C, Two signalling cascades mediated by metabolic transcription factors SREBPs and ChREBPs. D, Example of suppression of fatty liver and atherosclerosis by NTM in *ldlr^−/−^* mice fed a HFD (adapted from Liu *et al*, JAHA, 2013). See text for details

The encounter between microbial agents (such as LPS) or host endogenous irritants (also named “danger signals” such as heat shock proteins, nucleic acids, nuclear proteins) and their pattern recognition receptors (such as TLRs) and cytosolic DNA sensors evokes robust signalling to the nucleus mediated by signal transducers.^51^, ^52^, ^53^ These signalling cascades originate at the extracellular and intracellular sensors and “splash” onto genes’ regulome (Figure 2A,C). Intracellular sensors, NOD receptors, inflammasomes and STING receptors are activated.^48^, ^54^, ^55^, ^56^, ^57^, ^58^, ^59^, ^60^

### 5.1 The NF‐κB transcriptional cascade: the linchpin of inflammatory response

The NF‐κB transcription factor family, the master regulators of immunity, are swept towards the nuclear regulome (see Figure 2A). This signalling cascade originates either at the TLRs, including the interleukin 1 receptor, or at other receptors. These receptors recognize cytokines, autoantigens or bacterial immunotoxins termed superantigens, and proteases in immune and non‐immune cells. Signalosomes facilitate the flow of signals to the nucleus. These supramolecular complexes include scaffolding provided by the caspase recruitment domain (CARD) containing the membrane‐associated guanylate kinase protein 1 (CARMA 1) in immune cells or CARMA3 in non‐immune cells.^61^, ^62^ The TNF receptor‐associated factor 6 provides a platform for the signalling machinery responsible for the phosphorylation, ubiquitination and proteasomal degradation of the physiologic inhibitors of NF‐κB, termed IκB.^63^, ^64^, ^65^ Their phosphorylation depends on IκB kinases (IKK) α and β that form a complex with the regulatory protein NEMO^66^ (Figure 2A). NEMO is positively regulated by CARMA1 or 3 signalosome and negatively controlled by the A20 protein, a dual function ubiquitin‐modifying enzyme. The inborn deletion mutation of NEMO's binding segment for A20 causes the constitutive activation of IKK, which underlies the autoinflammatory disease of skin and intestines^6^ (see above). Phosphorylation “primes” IκBα for subsequent ubiquitination.^67^ Dephosphorylation by phosphatases counteracts these signalling steps.^68^, ^69^ Hence, phosphatases (eg PP2A) dephosphorylate IKKβ, IκBα and RelA slowing down or accelerating the signalling cascades.

Importantly, K63 polyubiquitination plays a key regulatory role in expediting the NF‐κB signalling cascade, while two deubiquitinases, the A20 protein and tumour suppressor cylindromatosis (CYLD), slow the flow of signalling that destroys IκBs.^63^ The removal of these inhibitors unmasks NLS on NF‐κB family members. Exposing these “zip codes” for nuclear delivery prompts instant recognition by the nuclear transport shuttles, importins/karyopherins α and β. These shuttles ferry NF‐κB to the nucleus as discussed below. In the nucleus, NF‐κB family members recognize the regulatory elements that control the expression of a large number of genes encoding the mediators of inflammation (see Table 2 that lists the mediators of inflammation encoded by the genes regulated by the transcription factors. They require nuclear transport shuttles targeted by nuclear transport modifiers abbreviated NTMs—see below).

**Table 2:** Genes encoding mediators of inflammation regulated by transcription factors that require nuclear transport shuttles targeted by NTMs

Mediators of inflammation	Transcription factor (MW/kDa)											
	NF‐κB1 p50 (48)	STAT‐1 (87)	AP‐1 c‐Jun (36)	Rel A (60)	NF‐κB2 p52 (49)	c‐Rel (68)	nSREBP1 (51)	NFATc4 (96)	NFATc1 (77)	NFATc2 (100)	NFATc3 116)	NRF‐2 (68)
Nuclear targeting motif	mNLS	uNLS	bNLS	mNLS	mNLS	mNLS	bHLH	mNLS	mNLS	mNLS	mNLS	mNLS
**Cytokines and growth factors**												
IL‐1a	+	+	+	+	+							
IL‐1b	+		+	+	+	+		+	+	+	+	
IL‐2	+		+			+		+	+	+	+	
IL‐6	+		+	+	+	+		+	+	+	+	
IL‐9	+	+	+	+		+		+	+	+	+	
IL‐10	+	+		+	+	+		+	+	+	+	+
IL‐12p40	+	+		+	+							
IL‐12p70	+	+			+							
IL‐13	+	+	+	+		+		+	+	+	+	
IL‐15	+					+						
IL‐17	+	+	+				+	+				
IL‐18/IGIF	+	+	+				+					
Ifng	+	+		+	+		+					
Lif	+	+	+	+	+	+	+	+	+	+	+	+
Lta	+	+	+	+	+	+	+	+	+	+	+	+
Tnf	+	+	+	+	+	+	+	+	+	+	+	+
Spp1	+	+	+									
G‐csf/Csf3	+	+	+	+		+	+	+	+	+	+	
M‐csf/Csf1	+	+	+	+	+	+	+	+	+	+	+	
Gm‐csf/Csf2	+	+	+	+	+		+					
Vegf	+	+	+				+					
**Chemokines**												
Ccl2/MCP‐1	+	+	+	+	+	+						
Ccl3/MIP‐1α	+	+	+		+							
Ccl4/MIP‐1β	+	+	+									
Ccl5/RANTES	+		+	+	+	+						
Ccl7/MCP‐3	+	+	+	+	+	+						
Ccl8/MCP‐2	+	+		+	+		+	+	+	+	+	
Ccl17/TARC	+			+	+	+						
Ccl19/MIP‐3β	+			+			+					
Ccl20/MIP‐3α	+			+	+	+						
Ccl22/MDC	+		+		+							
Ccl24/MPIF‐2	+			+	+	+		+	+	+	+	
Cx3cl1/ABCD‐3	+		+	+	+		+					
Cxcl2/MIP‐2α	+			+	+	+						
Cxcl5/LIX	+	+		+	+	+						
Cxcl9/MIG	+			+	+	+	+					
Cxcl10/IP‐10	+	+	+	+	+	+	+					
Cxcl11/IP‐9	+	+	+	+		+						
**Receptors/Transporters**												
Cxcr5/CD185	+	+	+	+	+	+	+					+
Ccr2/CD192	+	+	+				+					
Ccr3/CD193	+	+	+									
Ccr7/CD197	+	+	+			+						
Ccr10/GPR2	+	+	+				+	+	+	+	+	
IL‐6ra	+	+	+		+							
IL‐6st	+	+		+	+	+						
Tnfrsf1b	+	+	+	+	+	+						
Abcf1	+	+	+	+	+		+	+	+	+	+	+

Note

Analysis was performed in silico using The UCSC Genome Browser (The UCSC Genome Bioinformatics). “+”—the transcription factor–specific binding site is present in the gene promoter. mNLS—monopartite nuclear localization sequence; bNLS—bipartite nuclear localization sequence; uNLS—unconventional, structural or dimer‐specific nuclear localization sequence; bHLH—basic helix‐loop‐helix leucine zipper motif (adapted from DiGiandomenico *et al*, PLoS ONE, 2014).

### 5.2 The AP‐1 transcriptional cascade

The Activator Protein 1 heterodimer, made of cFos and c‐Jun, comprises another proinflammatory transcriptional cascade dependent on the Jun N‐terminal kinases (JNK) 1, 2 and 3.Their activity is regulated by JNK stimulatory phosphatase 1.^70^ The transcription factor cFos displays bipartite NLS recognized by importin α.^71^, ^72^ In immune cells, the signalosome CARMA1 regulates JNK 2 and the transcription factor c‐Jun in addition to regulating the NF‐κB signalling pathway.^73^ In this context, the selective ablation of the NF‐κB pathway with the transgenic degradation‐resistant IκBα mutant enhances the late signalling mediated by the AP‐1 heterodimer.^74^ A similar diversion may likely occur with signalling pathway‐selective inhibitors. Thus, the selective blockade of one signalling pathway allows other cascades to gain strength. It remains to be determined whether this “diversion rule” applies to the new classes of kinase inhibitors that target Janus kinases (JAKs) and the Bruton tyrosine kinase (BTK; see below) while having an enhancing effect on other proinflammatory transcriptional cascades.

### 5.3 The NFAT transcriptional cascade responds to calcium fluxes

The nuclear factor of activated T cells (NFATs) subfamily is comprised of four distinct calcium/calcineurin‐regulated transactivators that recognize genes encoding at least 16 mediators of inflammation (see Table 2). The fifth member is the osmotic stress‐dependent NFAT5.^75^ NFATs are constitutively phosphorylated by “priming kinase,” dual‐specificity tyrosine phosphorylation‐regulated kinase 1A, GSK‐3β, casein kinase‐1, p38 mitogen‐activated kinase and JNK. In response to the activation of Ca^2+^‐ and the calmodulin‐dependent serine/threonine phosphatase, calcineurin, NFATs are dephosphorylated.^76^, ^77^ Dephosphorylation of nearby serine/threonine residues unmasks NLS rendering NFATs eligible for nuclear transport.^78^, ^79^ Excessive expression of “Down Syndrome Critical Region (DSCR)‐1” counteracts the action of calcineurin, thereby averting nuclear translocation of NFAT in endothelial, cardiac and neuronal cells. Calcineurin/NFAT signalling cascade plays a key role in inflammation‐associated endothelial cell activation and angiogenesis.^75^

### 5.4 The STAT1 transcriptional cascade responds to interferon γ

This cascade is initiated by IFN γ interacting with its cognate receptor to activate JAK1 and JAK2 kinases. In turn, STAT1, the main transactivator of *Infγ*, is phosphorylated and translocated to the nucleus to activate several proinflammatory genes (see Table 2).^80^ This process is regulated by physiologic suppressors of cytokine signalling, SOCS1 and SOCS3, that control this receptor‐proximal step (see below). The nuclear transport of phosphorylated STAT1 is dependent on importin α5.^81^ A nuclear protein tyrosine phosphatase is required for the rapid nuclear export of STAT1.^82^

We termed STAT1, along with NF‐κB, AP‐1 and NFAT, “stress‐responsive transcription factors” (SRTFs), since they are activated by a mix of agonists imitating proinflammatory irritants.^45^

### 5.5 The Nrf 2 transcriptional cascade regulates oxidative stress, DNA sensors and inflammasomes

Oxidative stress, an integral part of inflammatory response,^83^ is sensed by the cytoskeleton‐based, Kelch‐like protein (Keap 1) (Figure 2A). Keap 1 is linked to the oxidative stress‐responsive transcription factor, the Nuclear factor erythroid 2‐related factor 2 (Nrf2). Its nuclear transport is dependent on importin α5.^84^, ^85^ In addition to the oxidative stress‐related function of Nrf2, this transactivator can recognize the regulatory elements of the six mediators of inflammation (see Table 2) while also functioning as a negative regulator of antiviral cytosolic DNA sensing. Significantly, Nrf2 positively regulates inflammasomes during metabolic inflammation exacerbating atherosclerosis while not altering lipid metabolism.^86^, ^87^, ^88^

## 6 INFLAMMASOMES: THE CRADLE OF CONSTITUTIVE INFLAMMATION

Inflammasomes comprise the nucleotide‐binding leucine‐rich repeat‐containing protein (NLRP). This protein regulates its partner, termed the adaptor protein apoptosis‐associated speck‐like protein, containing a CARD (ASC). They assemble upon a stress‐induced change in intracellular K^+^, Ca^2+^ and cyclic AMP concentrations to activate caspase 1.^60^, ^89^, ^90^ Prostaglandin E2 inhibits NLRP3 inflammasome activation.^91^ Caspase 1 processes members of the IL‐1 family, IL‐1 and IL‐18, that bypass the classic secretory pathway.^90^, ^92^ The gain–of‐function mutations of the genes encoding the regulators of caspase 1 underlie the constitutive inflammation mediating an autoinflammatory disease known as FMF along with other CAPS afflicting people of Middle Eastern ancestry, all replicated in a murine model.^2^, ^93^ Moreover, activated inflammasomes initiate pyroptosis, an inflammatory form of cell death that terminates intracellular pathogen proliferation.^60^, ^89^ The autophagy gene encoding ATG16L1 controls inflammasome activation.^94^ Strikingly, the initiators of metabolic inflammation producing atherosclerosis (cholesterol crystals) and gout (uric acid crystals) activate NLRP3 inflammasomes.^95^, ^96^

## 7 METABOLIC TRANSCRIPTIONAL CASCADES: THE DRIVERS OF METABOLIC INFLAMMATION

Metabolic inflammation, also termed “metaflammation,” is caused by metabolic stress due to overfeeding^97^ or accumulation of metabolites resulting from inborn metabolic errors that cause either gout^98^ or familial hypercholesterolaemia.^99^ At least two cascades of metabolic transcription factors, the sterol regulatory element‐binding proteins (SREBP) 1a, 1c and 2,^100^ and the carbohydrate response element‐binding proteins (ChREBP) α and β^101^ (Figure 2C) underlie metabolic inflammation.

### 7.1 The SREBPs transcriptional cascade maintains cholesterol homeostasis

SREBPs, members of the evolutionary conserved basic Helix‐Loop‐Helix Leucine Zipper family, are the master regulators of cholesterol, triglyceride and fatty acid synthesis.^102^ When a cell's cholesterol level drops below 5% of membrane lipids, the interaction between the endoplasmic reticulum (ER) membrane adaptor protein INSIG and the sterol sensor SCAP is weakened. This allows COPII, a vesicle coat protein, to translocate the SCAP/SREBP complex from the ER to the Golgi apparatus for proteolytic processing by site‐1 protease (S1P) and site‐2 protease.^103^ Subsequently, the processed SREBPs are dispatched to the nucleus by importin β1 (see Figure 2C).^104^ In the nucleus, SREBPs activate their own genes through a feed‐forward loop, as well as over 30 genes that encode the enzymes and binding proteins involved in the synthesis of cholesterol, triglyceride and fatty acids (eg *Srebf1*,* Srebf2*,* Hmgcr*,* Fasn*,* Acly*,* Pcsk9*,* Npc1l1*, see Figure 2C).^105^ Three protein products of these SREBPs‐regulated genes constitute the therapeutic targets of statins (HMGCR), monoclonal antibody (PCSK9) and ezetimibe (NPC1L1).^103^, ^106^, ^107^, ^108^ Moreover, SREBP1a induces the gene encoding inflammasome constituent NLRP1a^109^ (see above).

Under certain pathologic conditions, SREBP1c is activated either by insulin via the nuclear receptor LXRα and the transcription factor C/EBPβ,^110^ the CREB‐regulated transcription coactivator 2^111^ or by the vascular endothelial growth factor, a key inducer of angiogenesis and vascular permeability.^112^, ^113^, ^114^ Alternatively, the unfolded protein response associated with ER stress activates the SREBPs cascade initiated by Caspase 2‐mediated cleavage of the Golgi S1P protease.^115^ Irrespective of the mode of SREBP1 and SREBP2 activation, their common, importin β1‐mediated, nuclear transport is controlled by NTM,^116^ as discussed below.

### 7.2 The ChREBPs transcriptional cascade responds to hyperglycaemia

Elevated glucose initiates another cascade that activates the metabolic transcription factors, carbohydrate regulatory element‐binding proteins (ChREBPs) α and β. These transactivators regulate the expression of genes involved in glycolysis, lipogenesis and gluconeogenesis^101^ (see Figure 2C). The nuclear transport of ChREBPs depends on its dephosphorylation by the protein phosphatase PP2A that is activated by Xylulose 5‐Phosphate. Dephosphorylation of nearby serine/threonine residues within the basic helix‐loop‐helix site unmasks the NLS rendering of ChREBPs eligible for nuclear transport.^117^ This transport is also inhibited by NTM.^116^ In the nucleus, SREBP1c and ChREBP cooperatively induce glycolytic and lipogenic genes (*Gk*,* Pfk1*, *Pfkfb1*,* Aldob*,* Lpk*,* Acly*,* Acc*,* Fasn*,* Scd*).^118^ Deficiency of ChREBPs induces glycogen synthesis while reducing triglyceride formation. Thus, ChREBP's action results in shunting excess carbohydrates towards potentially harmful triglycerides instead of physiologically useful glycogen stores.^119^ Ultimately, the elevated glucose and triglycerides constitute the “deadly combination” leading to metabolic syndrome,^120^ the prevalent example of obesity, fatty liver, insulin resistance underlying Type 2 diabetes and atherosclerosis, all mediated by metabolic inflammation.

### 7.3 Other metabolic transcription factors

Metabolic inflammation also depends on the extended family of nuclear receptors comprised of three branches: (a) steroid hormone receptors exemplified by glucocorticoid and oestrogen receptors; (b) metabolite‐activated transcription factors, which form an ultimate complex with the retinoid X receptor^121^; and (c) the so‐called orphan receptors without required or identified ligands. Other metabolic transcription factors are known as hepatocyte nuclear factors (HNF) that are expressed in the liver, kidney and pancreatic islets.^122^ They regulate the insulin gene and other genes involved in the transport of glucose and metabolism (*Glut2*,* Aldob*,* Gapdh*,* Lpk*).

Cumulatively, metabolic inflammation, the main mechanism of atherosclerosis and its cardiovascular complications, Type 2 diabetes and fatty liver, depends on four classes of transcription factors (SREBPs, CHREBPs, nuclear receptors and HNF). The precise role of the NF‐κB pathway therein awaits elucidation (see below: Metabolic Inflammation of the Liver: From Steatosis to Steatohepatitis to End‐Stage Liver Disease).

## 8 PHYSIOLOGIC BRAKES ON INFLAMMATORY RESPONSE: STEROIDS AND RESOLVINS

Glucocorticoids, as exemplified by cortisol produced in the cortex of the adrenal gland, follow catecholamines (norepinephrine and epinephrine, the “fight or flight hormones”) in the general adaptation syndrome to stress.^123^ Glucocorticoids freely cross the cell membrane and bind to their cognate nuclear receptor in the cytoplasm. The complex is then transported to the nucleus by importin α7.^124^ Importin α7 is not targeted by NTM.^125^ Hence, the anti‐inflammatory actions of glucocorticoids and NTM are not mutually exclusive and can potentially synergize. In the nucleus, the glucocorticoid receptor binds to the glucocorticoid response elements while also downregulating several proinflammatory genes.^126^, ^127^, ^128^

Besides endogenous cortisol, the other physiologic lipid derivatives, resolvins, control the inflammatory response, accelerating its resolution. Resolvins encompass the three families of lipid mediators limiting innate immune responses and promoting microbial clearance displaying a cytoprotective effect in microbial inflammation.^129^ One of these resolvins inhibits the ER stress‐induced apoptosis of liver cells by suppressing SREBP‐1 expression and caspase 3 activity.^130^

## 9 SUPPRESSORS OF CYTOKINE SIGNALLING (SOCS)

Cytokines bind to their cognate receptors, for example IFN γ receptor (Figure 2A), and then initiate the proinflammatory signals mediated by both a family of JAKs and the transcription factors STAT1, STAT3, STAT4, STAT5 and STAT6.^80^ In response to cytokine‐evoked proinflammatory signals, the aforementioned SOCS1 and SOCS3 are rapidly expressed. They latch on their targets and destroy them while being also annihilated in this act through the ubiquitinylation and subsequent proteolysis by proteasomes.^131^ The receptors for cytokines and chemokines, the major mediators of inflammation, are the intracellular targets of SOCS1 and SOCS3 (see Table 2). SOCS 1 and 3 also target JAKs.

## 10 OTHER PHYSIOLOGIC CHECKPOINT REGULATORS

The other physiologic intracellular “checkpoint” regulators in transcriptional cascades include the IL‐1 receptor‐associated kinase (IRAK)‐M, the inhibitors of NF‐κB (IκB) (see above), the A20 protein, tumour suppressor CYLD, and the Caspase and Receptor Interacting Protein Adaptor with Death Domain (CRADD/RAIDD). They limit the duration and strength of the proinflammatory signalling pathways emanating from TLRs, cytokine receptors and protease‐activated receptors (PARs) in immune and non‐immune cells, for example endothelial cells.^132^ SH2‐containing inositol‐5‐phosphatases (SHIP and SHIP1) counteract the signalling events based on tyrosine phosphorylation that are evoked by Fcγ receptors. SHIP1‐deficient mice display spontaneous airway inflammation as well as an increased sensitivity to allergen‐induced airway inflammation.^133^

### 10.1 A20: the guardian of gut–microbiome homeostasis

The genetic ablation of A20, a dual ubiquitin modifier enzyme, wreaks havoc in the homeostatic control of the gut microbiome, causing lethal microbial inflammation mediated by the MyD88‐dependent signalling pathway in A20‐deficient pups.^134^, ^135^ In humans, the escape from the physiologic anti‐inflammatory action of A20 in individuals born with the deletion of the NEMO carboxy‐terminal segment underlies the constitutive inflammation of the skin and intestines due to continually active NF‐κB signalling causing this new example of autoinflammatory disease.^6^

### 10.2 CRADD: the regulator of CARMA signalosome

We found a new regulatory function for CRADD/RAIDD as another physiologic suppressor of inflammation.^136^ CRADD/RAIDD targets a key signal transducer, cytoplasmic B‐cell lymphoma/leukaemia 10 (Bcl10), a signalling effector of CARMA 1 in immune cells or CARMA3 in non‐immune cells. BCL10 is a positive regulator of NEMO in the NF‐κB signalling cascade. The intracellular delivery of recombinant cell‐penetrating (CP)‐CRADD restored endothelial barrier function and suppressed the production of inflammatory mediators IL‐6 and monocyte chemoattractant protein‐1.^137^ CRADD's target, Bcl10, is essential for the development of atherosclerosis and abdominal aortic aneurysms, the bulging weak spots in the vascular wall that can dissect and rupture in ApoE‐deficient mice. These mice were fed a high‐fat diet (HFD) and were stimulated with the angiotensin receptor ligand, angiotensin II.^138^ Thus, CP‐CRADD offers a potential countermeasure, in addition to the currently used angiotensin receptor blockade,^139^ for rapid control of aortic aneurysms, a life‐threatening complication with 50% mortality.

## 11 THE GLOBAL GENOMIC REPROGRAMMING VERSUS ONE‐GENE–ONE‐PROTEIN TARGET CONCEPT

The fundamental approach to containing inflammation rests on its transcriptional paradigm.^45^ Transcription factors activate or repress numerous genes underlying inflammation‐mediated diseases in immune and non‐immune cells (see Table 2). The single protein products of these induced genes, for example TNF α, are a therapeutic target of a monoclonal antibody, while another product, the signal transducer COX‐2, is inactivated by aspirin, along with other Non‐Steroidal Anti‐Inflammatory Drugs ( NSAIDs). Targeting a single mediator of inflammation has advanced the treatment of several inflammatory diseases.^140^, ^141^, ^142^, ^143^ TNFα induces signalling cascades through its two cognate receptors (proinflammatory and proapoptotic). The proinflammatory receptor evokes the NFĸB signalling pathway that regulates the expression of several inflammatory mediators.^144^ Whereas the expression of these mediators can be suppressed by targeting TNFα, other inflammatory mediators generate signals through their cognate receptors that also evoke the NFĸB signalling pathway. Nevertheless, targeting TNFα attenuates autoimmune inflammation in some diseases settings while reactivating a latent tuberculosis infection.^145^ Thus, the one‐gene‐one‐protein target strategy misses many other inflammatory mediators that are encoded in the human genome and expressed concomitantly with a targeted single protein. Among them, the IFNγ and the pleiotropic cytokine IL‐6 signal to the nucleus through their cognate receptors activating their respective JAK‐STAT signalling pathways. In contrast to anti‐TNFα monoclonal antibody, anti‐inflammatory glucocorticoids downregulate several proinflammatory genes.^126^, ^127^, ^128^, ^146^ Still, the anti‐inflammatory glucocorticoid function is counteracted by proinflammatory cytokines, TNFα and IL‐1β.^147^ Moreover, the metabolic side effects of glucocorticosteroids such as hyperglycaemia, hyperlipidemia and osteoporosis are of concern.^146^, ^148^, ^149^ These limitations and other drawbacks of current anti‐inflammatory agents inspired us to search for alternative, wide‐reaching, anti‐inflammatory measures.

## 12 INTRACELLULAR PROTEIN THERAPY WITH CELL‐PENETRATING RECOMBINANT SOCS3: A FACILE ALTERNATIVE TO GENE THERAPY

We sought to limit the availability of transcription factors responding to proinflammatory insults. In the cytoplasm of an “inflamed” cell, the physiologic SOCS 1 and 3 control the availability of transcription factors known as STAT 1 and 3.^80^ These transactivators regulate the genes encoding multiple cytokines and chemokines including IFNγ, IL‐2, IL‐4, IL‐6, IL‐7, IL‐15, IL‐17, the leukaemia inhibitory factor, leptin and the granulocyte‐colony stimulating factor (G‐CSF).^150^ The cytoplasmic tails of their receptors as well as the signal transducers, JAK, are targeted by SOCS1 and SOCS3 for ubiquitin‐mediated proteasomal degradation while simultaneously being destroyed by the same mechanism.^131^, ^151^, ^152^ SOCS1 and SOCS3 also impede the transcriptional cascades triggered by TLRs.^153^

**Figure 3:**
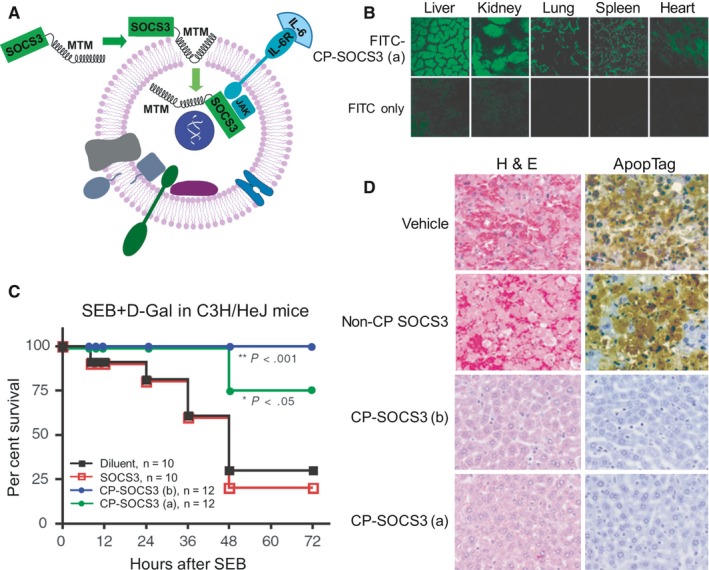
Intracellular protein therapy with cell‐penetrating (CP) recombinant SOCS3 in metabolically compromised mice challenged with superantigen SEB. A, Conceptual depiction of intracellular delivery of CP‐SOCS3 crossing membrane phospholipid bilayer in energy‐independent process that bypasses endosomal compartment. MTM—membrane‐translocating motif. CP‐SOCS3 targets cytoplasmic tail of IL6 receptor (IL6R) and JAK. B, In vivo delivery of FITC‐labelled CP‐SOCS3 to major organs; (a): CP‐SOCS3 variant with MTM located at the NH_2_ terminus of SOCS3. C, Improved survival of mice challenged with superantigen SEB and treated with CP‐SOCS3; (b): CP‐SOCS3 variant with MTM located at the COOH terminus of SOCS3. D, Inflammation‐driven haemorrhagic necrosis and apoptosis of the liver cells in SEB‐challenged mice is suppressed by treatment with CP‐SOCS3 (a) and (b) (adapted from Jo *et al*, Nat. Med. 2005)

The kamikaze‐like self‐destructive activity of short‐lived SOCS1 and SOCS3 against cytokine receptors and JAKs motivated us to design and engineer recombinant cell‐penetrating orthologues of SOCS1 and SOCS3 (CP‐SOCS1 and CP‐SOCS3). By bioengineering recombinant cell‐penetrating SOCS1 and SOCS3, we introduced a new class of anti‐inflammatory intracellular protein therapy.^21^, ^154^, ^155^ Within minutes, CP‐SOCS3 replenished the intracellular stores of SOCS3 that would be consumed during acute liver inflammation (see Figure 3). In contrast, gene transfer‐produced proteins experience days‐long delays in attaining sufficient levels of the functional intracellular proteins that cannot be controlled.^156^ Surprisingly, CP‐SOCS3 is less prone to degradation, thereby extending its intracellular half‐life to 6.2 hours, while CP‐SOCS3 with a deleted SOCS box persists for at least 29 hours, compared to the 42‐minute half‐life of the endogenous form of SOCS3. In an in vivo model of fulminant microbial inflammation of the liver, recombinant CP‐SOCS3 dramatically protected hepatocytes and other cells from apoptosis and haemorrhagic necrosis, thus supporting a new concept of intracellular protein therapy for acute liver injury and other rapidly progressing, inflammatory disorders^21^, ^154^, ^155^ (see Figure 3).

## 13 CP‐SOCS1‐ AND SOCS1‐DERIVED PEPTIDES

Suppressor of cytokine signalling (SOCS)‐1 is a classic negative feedback inhibitor of the IFN‐γ‐induced JAK‐STAT pathway that controls potentially harmful intracellular signalling during excessive inflammation.^157^ Several inflammatory diseases are mediated by uncontrolled IFN‐γ signalling. SOCS1‐deficient mice display multiple organ injury with a striking lymphocyte depletion that is abrogated by the genetic ablation of the IFN γ gene.^158^ Moreover, SOCS1 quenches the activation of LPS‐induced TLR4 signalling by binding to the MyD88‐like adaptor MAL/TIRAP and inducing its ubiquitin‐mediated degradation. Hence, SOCS1 attenuates MAL‐dependent phosphorylation and the transactivation of NF‐κB RelA.^159^ Therefore, we engineered a recombinant cell‐penetrating SOCS1 (CP‐SOCS1) to target the IFN‐γ signalling pathway.^154^ The structure‐function analysis of SOCS1 indicates that multiple domains perform a distinct function. The amino terminal Kinase Inhibitory Region (KIR), also present in SOCS3 but not in the other known members of the SOCS family of proteins,^160^, ^161^, ^162^ inhibits JAKs activity. The centrally located SH2 domain in SOCS1 (and SOCS3) binds the phosphorylated tyrosine residues on JAK proteins and cytokine receptors. Finally, the carboxy‐terminal SOCS box serves as an E3 ubiquitin ligase that targets signalling proteins for proteasomal destruction. Together with the NH_2_‐terminal PEST domain, they contribute to the rapid turnover of SOCS proteins.^163^ We showed that loss of the PEST domain does not affect CP‐SOCS1 inhibitory potency, while the double knockout, CP‐SOCS1/ΔPEST.SB, consistently revealed greater activity.^154^ The increased activity of CP‐SOCS1/ΔPEST.SB might be due to the loss of the PEST domain, which was shown to increase protein turnover.^163^ Hence, its deletion leads to the increased intracellular stability of CP‐SOCS1/ΔPEST.SB.

It is apparent that the multidomain structure of SOCS1 and SOCS3 allows for a broader spectrum of anti‐inflammatory activities than the KIR domain. Its target, the JAKs, display an intrinsically complex structure comprising the 4.1, ezrin, radixin, moesin SH2 (Src homology 2) pseudokinase and kinase domains.^164^ The short 16‐amino acid peptide mimetics of SOCS1 KIR domain were designed with an attached lipophilic group to penetrate the cell membrane and target JAKs.^165^ In a murine model of experimental allergic encephalomyelitis, SOCS1‐KIR peptide alleviated paraplegia and other signs of the cellular infiltration of the central nervous system. A similar SOCS1‐KIR peptide was tested topically in inflammatory eye disease, experimental autoimmune uveitis.^166^ In both studies, the suppression of IFNγ and IL‐17 was noted. Whether a broader scope of the anti‐inflammatory action of CP‐SOCS1 and CP‐SOCS3 can be substituted with short SOCS1‐KIR peptides requires a more detailed analysis of their bio‐distribution, half‐life and potential off‐target effects.

## 14 CONVERGENCE OF PROINFLAMMATORY TRANSCRIPTIONAL CASCADES AT THE NUCLEAR ENVELOPE

The signalling cascades of proinflammatory transcription factors converge in the cell's nuclear territory. There, the nuclear transport system constitutes the pivotal checkpoint for several transcriptional cascades (Figure 2A,C). These signalling pathways encompass the representatives of at least five families of human transcription factors responsible for expressing a multitude of inflammatory mediators (Table 2).^36^, ^51^

Crossing the nuclear envelope is controlled by the nuclear pores, complex molecular sieves that allow the free movement of cytoplasmic proteins smaller than 40 kD to and from the nucleus.^167^, ^168^ The small transcription factors essential to cell survival and maintenance such as SFRS9 (serine/arginine‐splicing factor 9) also known as Srp30c (~27 kD) have a “free pass.” Remarkably, SFRS9 activates the expression of more than 150 genes, among them 50 genes that encode other transcription factors.^116^, ^169^ In contrast, larger transcription factors (or their dimers), above the 40‐60 kD range, require cytoplasmic/nuclear “shuttles.” These shuttles, termed importins/karyopherins α and β, recognize the nuclear “zip codes” provided by the NLS on transcription factors. Upon binding their cargo, importins α form a tandem with importin β1 that guides the complex to the nuclear pore through a GTP/GDP gradient controlled by Ran GTPase.^167^, ^170^, ^171^, ^172^ NTMs dismantle this nuclear translocation process. Beyond the nuclear transport checkpoint, transcription factors’ DNA accessibility is regulated by histone acetylation and deacetylation, as shown with the Foxp3 and glucocorticoid receptor, and by topoisomerase 1.^173^, ^174^, ^175^

## 15 THE NUCLEAR TRANSPORT CHECKPOINT IS A TARGET FOR WIDE‐RANGING ANTI‐INFLAMMATORY THERAPY

The design and facile application of cell‐penetrating NTMs gave rise to a wide‐ranging experimental therapy in preclinical models of microbial, autoimmune, metabolic and physical (post‐traumatic) inflammation, as we will discuss below.

The prototypical NTM, the SN50 peptide, comprises the NLS region derived from human NF‐κB1 as a potential competitor of the nuclear transport of NF‐κB family members, thereby averting the activation of NF‐κB‐regulated genes that encode the mediators of inflammation (see Table 2). We enabled NLS delivery across the cell membrane of immune and non‐immune cells by attaching the membrane‐translocating motif. It is based on the signal sequence hydrophobic region (SSHR) of the human fibroblast growth factor 4.^176^, ^177^ This hydrophobic “leading edge,” attached to peptides or proteins of choice, penetrates the plasma membrane by directly traversing the phospholipid bilayer without involving the chirally specific receptor/transporter mechanisms.^155^, ^178^ Importantly, the hydrophobic leading edge stealthily bypasses the endosomal compartment, thereby avoiding the potential degradation of the attached cargo by lysosomal proteases. In contrast, the alternative cell‐penetrating peptide motifs derived from HIV Tat or Antennapedia proteins can accumulate in the endosomal compartment. These motifs are potentially immunogenic, being derived from non‐human proteins.^178^, ^179^

## 16 A SINGLE NTM DISMANTLES THE FOUR PROINFLAMMATORY TRANSCRIPTIONAL CASCADES THAT TRIGGER THE GENOMIC STORM IN SEVERE INFLAMMATION

**Figure 4:**
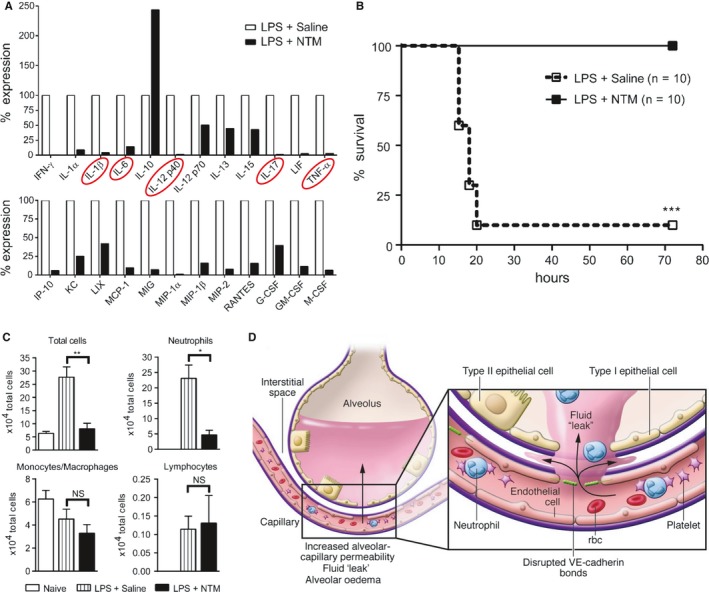
Broad anti‐inflammatory action of NTM (cSN50.1 peptide) in mice challenged with bacterial endotoxic lipopolysaccharide (LPS). A, NTM treatment reduced plasma levels of 23 proinflammatory mediators and increased blood content of anti‐inflammatory cytokine, IL‐10. Red circles point to cytokines currently targeted by therapeutic monoclonal antibodies. B, NTM treatment improved survival in C57BL/6 mice challenged with lethal dose of LPS (800 µg) administered through intraperitoneal injection (A and B). C, NTM treatment prevented migration of neutrophils and monocytes to the lungs in LPS‐induced acute lung injury (ALI) (A, B and C adapted from DiGiandomenico *et al*, PLoS ONE, 2014). D, Conceptual depiction of microvascular endothelial injury in the lungs in ALI (adapted from Matthay *et al*, JCI, 2012)

Significantly, the first NTM (SN50 peptide) suppressed not only nuclear translocation of the master regulator of immunity, NF‐κB, but also the nuclear transport of AP‐1 and NFAT that cooperatively induce the expression of the main immunoregulatory and proinflammatory cytokine IL‐2.^43^, ^44^ Our initial evidence that *Il2* gene expression can be controlled in human T cells by the inhibition of nuclear import at the level of recognition of NLS displayed by NF‐κB, AP‐1 and NFAT^43^ was extended to LPS‐stimulated primary bone marrow‐derived macrophages. Among the 84 genes studied, at least 37 were suppressed by NTM (cSN50.1 peptide). The suppressed genes encode the mediators of inflammation encompassing cytokines and growth factors, chemokines and their receptors. Importantly, NTM did not suppress the expression of five housekeeping genes nor altered cell viability.^51^ In systemic severe microbial inflammation exemplified by LPS‐induced lethal shock, the cSN50.1 peptide afforded 75% protection when administered after LPS exposure. This NTM potently inhibited 23 out of 26 LPS‐induced proinflammatory cytokines, chemokines and growth factors such as TNFα, IL‐1β, IL‐2, IL‐6, IL‐12, IL‐17 and IFNγ (Figure 4). Of note, IL‐2 is a potent inflammatory mediator disrupting the blood‐brain barrier and altering brain microcirculation that underlies vascular leak syndrome and brain autoimmune inflammation.^20^ Furthermore, suppression of IL‐17A is particularly significant in microbial and autoimmune inflammation.^180^ In neonatal sepsis, the recently identified axis comprising IL‐18/IL‐1R1/IL‐17 contributes to mortality.^181^

It is immediately apparent that each of 23 cytokines and chemokines that are cumulatively suppressed by a single NTM would require 23 separate cognate monoclonal antibodies for neutralization. Thus, one strategically deployed anti‐inflammatory agent, such as NTM, can substitute for multiple monoclonal antibodies directed against the diverse mediators of inflammation.

At this junction, we emphasize that the SN50 peptide is not exclusively an “NF‐κB inhibitor,” as inaccurately advertised by commercial peptide manufacturers and reported by their customers in numerous publications. Rather, SN50 and its congeners target a common nuclear transport shuttle, importin α5^125^ also named karyopherin α1 that recognizes NLS on multiple SRTFs.^22^, ^51^, ^52^, ^81^, ^125^ The next‐generation NTM, the cSN50.1 peptide, has the highest solubility (100 mg/mL) compared to 13 mg/mL of the prototypical SN50 peptide.^51^ The binding of the NTM NLS module to importin α5 was specific and of high affinity (*K*_D1_ = 73 and *K*_D2_ = 140 nmol/L) with a 2:1 stoichiometry, suggesting that NTMs interact with both, major and minor NLS binding pockets, on importin α5.^125^ Significantly, mice with importin α5 deficiency are viable and fertile and do not display any apparent morphological and behavioural defects.^182^

## 17 METABOLIC TRANSCRIPTIONAL CASCADE: A SURPRISING TARGET OF NTM

Having demonstrated that NTM suppressed the proinflammatory stress‐responsive transcription factors, we next sought to determine whether atherosclerosis, a prime example of metabolic inflammation (see below) also can be suppressed. In collaboration with Amy Major, we embarked on studying NTM treatment of LDL receptor‐deficient mice fed a HFD for several weeks, a murine model of the human disease familial hypercholesterolaemia.^183^ NTM reduced atherosclerotic plaque formation and macrophage infiltration in the coronary sinus^116^ (Figure 2D). Unexpectedly, NTM showed a dramatic reduction of cholesterol in the blood and liver of LDL receptor‐deficient mice. We also found that NTM (cSN50.1) reduces SREBP1 and SREBP2 nuclear translocation induced by lipid depletion of cultured cells.^116^ We were puzzled because SREBPs do not possess a classic NLS motif in contrast to NF‐κB and other proinflammatory stress‐responsive transcription factors. Instead, SREBPs have the highly conserved basic helix‐loop‐helix forming a dimer that binds to importin β1.^104^ Fortuitously, we found that NTMs also recognize importin β1. This second function vested in the SSHR motif of NTMs (SN50, cSN50 and cSN50.1 peptides) explains the mechanism for NTM interference with SREBP nuclear import.^116^ In contrast, the peptides representing linear or cyclized NLS interact only with importins α, specifically with Imp α5.^125^ We realized that NTM possessed a dual function. The first function, the inhibition of proinflammatory stress‐responsive transcription factors (NF‐κB, AP‐1, NFAT and STAT‐1) by competing with NLS‐binding site on importin α5, is consistent with our prior studies of microbial inflammation discussed above. The second function was identified by us in the non‐NLS part of NTM. This part inhibits the importin β1 binding site for the basic helix‐loop‐helix segment of metabolic transcription factors, SREBPs. Indeed, the metabolic transcription factors SREBP1 and SREBP2, activated by an autoregulatory feed‐forward loop, were reduced by NTM in the liver. Likewise, six other genes regulated by SREBPs, including HMG‐CoA reductase, the target of statins, were suppressed as well as Nieman‐Pick C1‐like 1 protein, a key enteropatic cholesterol absorption receptor and a target for ezetimibe. In contrast, two genes encoding proteins responsible for cholesterol enterohepatic efflux (*Abcg5* and *Abcg8*) were not reduced.^116^ Thus, the SN50 family of cell‐penetrating peptides (SN50, cSN50 and cSN50.1) comprises the bi‐selective NTMs that bind both importin α5 and importin β1, thereby strategically targeting the nuclear transport checkpoint for SRTFs and metabolic transcription factors SREBPs and ChREBPs (Figure 2A,C).

## 18 OTHER NUCLEAR IMPORT INHIBITORS

The NF‐ĸB inhibitor structurally related to the antibiotic epoxyquinomycin was reported to inhibit the nuclear translocation of NF‐κB RelA (p65) in TNFα‐stimulated cells without information on the nuclear transport of other SRTFs.^184^ This inhibitor also reduced the expression of IL‐1β in human THP‐1 cells challenged with titanium dioxide particles and also reduced skin inflammation in a murine model of atopic dermatitis.^185^, ^186^ Ivermectin, a broad‐spectrum antiparasitic compound, was identified as an inhibitor of importin α/β nuclear import involved in the nuclear transport of some viral proteins.^187^ Its selectivity towards individual importins α remains unclear.

## 19 COMPARATIVE ANALYSIS OF ANTI‐INFLAMMATORY COUNTERMEASURES

**Figure 5:**
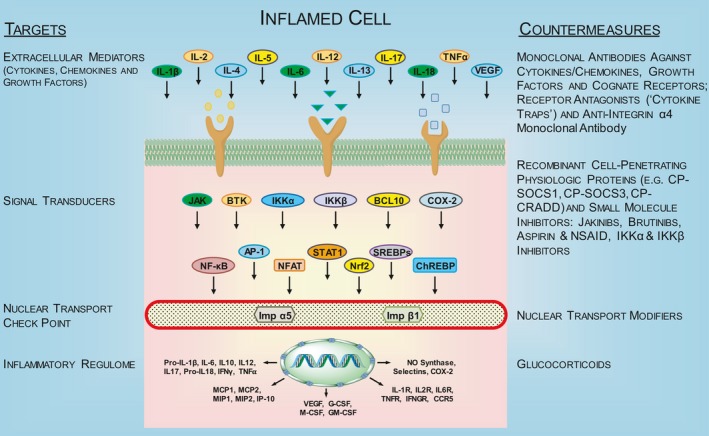
Anti‐inflammatory Strategies. Schematic diagram indicating four classes of targets of anti‐inflammatory intervention: extracellular mediators, signal transducers, nuclear transport checkpoint and inflammatory regulome

Diseases mediated by the six types of inflammation (Table 1) continue to pose a challenge to their prevention and therapy. The inflamed cell depicted in Figure 5 highlights the known intracellular and extracellular targets for anti‐inflammatory countermeasures that are discussed below.

## 20 INTRACELLULAR THERAPIES THAT TARGET SIGNAL TRANSDUCERS IN PROINFLAMMATORY SIGNALLING PATHWAYS

### 20.1 Classic anti‐inflammatory agents: Nonsteroidal Anti‐Inflammatory Drugs (NSAID's), steroids and methotrexate

Aspirin continues its preeminent role as an anti‐inflammatory remedy. The identification of its intracellular target COX2, a key enzyme in the synthesis of prostaglandins, led to the development of other NSAIDs with their known benefits, limitations and risks.^140^, ^141^ Of note, NTMs suppress COX2 expression and subsequent PGE2 production in inflamed cells.^188^ Another target of aspirin is IKKβ.^189^

The second major intracellular therapy of inflammation‐mediated diseases is based on the physiologic hormone cortisol that is produced in the adrenal cortex upon stimulation with the adrenocorticotropic hormone (ACTH) secreted by the pituitary gland.^126^, ^127^, ^128^, ^146^ Cortisol and its more potent analogs penetrate the cell membrane and bind to the glucocorticoid receptor belonging to the class of nuclear receptors described above. Through binding to distal enhancers whose chromatin accessibility is usually cell‐type specific,^128^ the glucocorticoid receptor reprogrammes the inflammatory regulome. After LPS‐induced genomic reprogramming, the glucocorticoid receptor produced a similar gene expression to the kind observed before the LPS challenge, while enhancing the expression of metabolic genes.^190^ Whereas glucocorticoids suppress the inflammatory regulome, they cause hyperglycaemia, hyperlipidemia, osteoporosis and immunosuppression. The untowards side effects of glucocorticoid therapy limit its utility in inflammation‐mediated diseases.^146^, ^148^, ^149^

Methotrexate is one of the mainstays of the therapy of autoimmune inflammation that mediates rheumatoid arthritis and psoriasis. This inhibitor of dihydrofolate reductase dampens the production of purines and pyrimidines, thereby interfering with DNA synthesis.^191^ Its putative mechanism of anti‐inflammatory action is linked to the formation of methotrexate polyglutamates and the inhibition of adenosine deaminase and AMP deaminase ultimately raising the level of adenosine, a compound known for its anti‐inflammatory action.^192^ As the mechanism of the anti‐inflammatory action of methotrexate remains incompletely understood,^193^ its use as a “broad‐spectrum anti‐inflammatory agent” in the large randomized clinical trial of cardiovascular diseases that are mediated by metabolic inflammation has failed.^194^

### 20.2 Inhibitors of kinases in proinflammatory transcriptional cascades

Three classes of kinases, BTK, IKKβ and JAKs, are polar signal transducers in the transcriptional cascades that deliver NF‐κB and STAT transcription factors to the nucleus (see Figure 2A). BTK is required for the activation of IKK and NF‐κB in B cells.^195^ Inborn deficiencies of BTK underlie X‐linked immunodeficiency.^196^ In turn, the activating mutations of JAK2 cause myeloproliferative diseases.^197^

IKKβ is targeted selectively by a cell‐penetrating peptide delivering the NEMO‐binding domain to disrupt the NF‐ĸB signalling pathway.^198^, ^199^ A modified version of this peptide termed the “sneaking ligand construct” was designed to target IKKβ in endothelial cells to ameliorate rheumatoid arthritis in experimental model.^200^

The BTK inhibitors (ibrutinib, acalabrutinib), IKKβ inhibitors (aspirin) and JAKs inhibitors (tofacitinib, ruxolitinib, baricitinib, lestaurtinib and pacritinib) are used to treat autoimmune and myelodysplastic diseases.^201^ Ibrutinib, introduced to treat chronic lymphocytic leukaemia, reduced plasma levels of 10 inflammation markers, mostly chemokines.^202^

As the JAK family comprises four members (JAK1, 2, 3 and TYK2), some inhibitors are mono‐selective while others target two or more members of the family. JAK inhibitors are effective in myeloproliferative diseases, rheumatoid arthritis, psoriasis and ulcerative colitis but not in Crohn's disease.^197^, ^203^ The homeostasis of NK cells and macrophages was most profoundly perturbed by JAK inhibitors with a longer lasting repression of IFN signature genes. Some of these changes persisted after the discontinuation of treatment.^204^ These findings are important as JAK inhibitors, also named Jakinibs, have significant side effects such as serious and opportunistic infections with *Mycobacterium tuberculosis*,* Herpes zoster*,* Cytomegalovirus*,* Pneumocystis jirovecii* pneumonia and other pneumonias.^197^ Thus, the suppression of autoimmune inflammation by Jakinibs may inadvertently promote the emergence of microbial inflammation. Moreover, recent reports also indicate the risk of thromboembolic events (deep vein thrombosis and pulmonary embolism).^205^

## 21 EMERGING ANTI‐INFLAMMATORY COUNTERMEASURES

As documented above, we expanded this group of intracellular anti‐inflammatory agents with the cell‐penetrating recombinant SOCS, CP‐SOCS3 and CP‐SOCS1, that are physiologic antagonists of the JAK‐STAT–mediated pathways (Figure 3). The second type of intracellular anti‐inflammatory agents, NTMs, dismantle the nuclear transport of four stress‐responsive transcription factors signalling cascades, including NFĸB, AP‐1, NFAT and STAT1, and two metabolic transcription factors signalling pathways SREBPs and CHREBPs (Figure 2A,C). NTM (cSN50.1 peptide) reduced plasma level of 23 out of 26 LPS‐induced proinflammatory cytokines and chemokines.^51^ It also suppressed the expression of nine genes that encode the key proteins involved in the synthetic pathways of cholesterol, triglycerides and fatty acids.^116^

### 21.1 The potential for “off‐target” effects of CP‐SOCS3 and nuclear transport modifiers

The targets for CP‐SOCS3 do not include only JAKs and cytoplasmic tails of multiple cytokine receptors (see above). CP‐SOCS3 potentially targets the intracellular segments of receptors for leptin, insulin, erythropoietin and G‐CSF.^152^ These targets may likely be affected upon chronic administration of CP‐SOCS3 rather than during the short‐term treatment of acute injury of the liver (eg fulminant hepatitis) 21 and other organs (eg acute respiratory distress syndrome [ARDS]). In contrast to the gene transfer therapy with SOCS3^156^ wherein the transferred gene product expression is uncontrolled, intracellular protein therapy with CP‐SOCS3 can be instantly stopped and its intracellular level would decline within 6 to 29 hours of the extended half‐life of recombinant CP‐SOCS3 variants.^155^ NTMs have an intracellular persistence not exceeding 180 minutes.^206^ As a bipartite peptide derived from the highly conserved segments of two human genes, prototypical NTM (SN50 peptide) was non‐immunogenic in rabbits following an adjuvant‐based immunization protocol. NTM's potential for immunosuppression was considered during the continuous 8‐week administration. No changes in blood cell numbers, including lymphocyte subsets, were detected in LDL receptor‐deficient mice fed a HFD, a model of familial hypercholesterolaemia (see below).^116^ The lack of apparent changes in blood cell counts was reassuring as NTM‐targeted importin β1 ferries SREBP2, which trans‐regulates the Notch pathway genes required for haematopoiesis.^207^ Furthermore, no signs of infection or impaired wound healing were observed during the 8‐week study with weekly surgical replacement of subcutaneous osmotic pumps.^116^ Thus, prolonged NTM administration did not seem to increase the susceptibility to environmental pathogens. These observations are consistent with the enhancement of bacterial clearance by NTM in a polymicrobial sepsis model.^52^

## 22 THE ANTI‐INFLAMMATORY ACTION OF THE TOPOISOMERASE 1 INHIBITOR

Others reported the anti‐inflammatory action of the topoisomerase 1 inhibitor camptothecin.^175^ However, the topoisomerase inhibitors‐based treatment of inflammation can activate a strong antiviral response dependent on the presence of viral oncogenes.^208^ Unleashed endogenous retroelements trigger the increased production of type 1 interferons leading to autoimmune inflammation.^209^ Camptothecin and its semisynthetic derivative irinotecan are associated with steatohepatitis and colitis.^210^, ^211^

## 23 EXTRACELLULAR THERAPIES THAT TARGET INFLAMMATORY MEDIATORS AND THEIR COGNATE RECEPTORS

### 23.1 Monoclonal antibodies, anakinra and cytokine traps

Georges Köhler and Cesar Milstein initiated a new era of immunology in 1975 by designing and characterizing a continuous hybridoma cell line producing a monoclonal antibody of immense diagnostic and therapeutic potential.^212^ They received the 1984 Nobel Prize in Physiology and Medicine. Jan Vilćek and his colleagues experimentally demonstrated the anti‐inflammatory action of the first anti‐TNFα monoclonal antibody.^142^ Patients with rheumatoid arthritis mediated by autoimmune inflammation showed significant improvement after treatment with the anti‐TNFα monoclonal antibody.^213^ Since that initial clinical study, published over 25 years ago, a growing number of at least 40 therapeutic monoclonal antibodies approved for clinical treatment of neoplastic and autoimmune diseases have emerged, including antibody‐drug conjugates, bispecific antibodies and glycol‐engineered antibodies.^214^, ^215^ Currently, 4175 active or completed clinical trials of monoclonal antibodies are registered worldwide, including 2764 in the United States.^201^ Among the latter, 2172 trials are conducted in patients with cancer.

Soluble receptor antagonists, for example Anakinra, and cytokines traps, for example Rilonacept and Etanercept, are alternatives to monoclonal antibodies to target extracellular mediators of inflammation.^216^, ^217^, ^218^

### 23.2 Monoclonal antibodies–induced autoimmune and microbial inflammation

The growing use of monoclonal antibodies in neoplastic, autoimmune and allergic diseases revealed the inherent risks and adverse effects of this unusually expensive therapy. For example, the cytotoxic T lymphocyte–associated protein 4 blocking antibody (ipilimab) induces hypophysitis in about 4% of treated patients who develop systemic manifestations of severe pituitary deficiency.^219^ A combination of ipilimab with the PD1‐blocking monoclonal antibody (nivolumab) causes adverse effects such as autoimmune inflammation–mediated colitis, encephalitis, hepatitis, nephritis, pneumonitis, thyroiditis, thrombotic thrombocytopenic purpura and the aforementioned hypophysitis. These side effects emerge during therapy or, within weeks to months after therapy.^220^, ^221^

Among these organ‐specific or systemic inflammatory disorders, two are perilously life‐threatening: fulminant myocarditis and the so‐called cytokine release syndrome with signs of microvascular endothelial injury manifested by ARDS and disseminated intravascular coagulation.^222^, ^223^ The term “cytokine release syndrome” is a misnomer since the cause of this complication, that is monoclonal antibody treatment, is known and cytokines are induced together with other mediators of inflammation rather than being “released” since they are usually not stored in secretory granules or vesicles.

Other monoclonal antibodies carry the risk of infections mediated by microbial inflammation. The anti‐TNFα monoclonal antibody–based therapies are linked to the reactivation of a latent infection with *Mycobacterium tuberculosis*.^224^ The treatment of multiple sclerosis with the monoclonal antibody natalizumab carries the risk of JC virus–associated progressive multifocal leukoencephalopathy that contributes to significant morbidity and mortality.^225^ Again, the suppression of autoimmune inflammation may reignite microbial inflammation.

## 24 A BRIEF INSIGHT INTO THE FIVE TYPES OF INFLAMMATION

### 24.1 Microbial inflammation

Microbial inflammation caused by a wide range of microorganisms can engulf any organ in the human body. The microbial pathogen–specific port of entry (known as organ tropism), propagation and spread are accompanied by a florid, and in some notable instances, persistent inflammatory response. Certain pathogens have hijacked the genes encoding inflammatory mediators to subvert host defences and propagate microbial inflammation.^226^ Pathogen‐directed antimicrobial therapy contains the cause of infection.

### 24.2 Microbial inflammation of the lungs due to gram‐negative bacteria

Multidrug‐resistant, Gram‐negative bacteria cause lung infections that have increased in hospitals throughout the United States and around the world. These infections lead to sepsis and septic shock, especially in immunocompromised hosts.^132^, ^227^ Patients with severe community–acquired pneumonia treated with macrolide antibiotics and aspirin had a 30‐day mortality of 15.5% as compared to 23.8% in macrolide only treated group.^228^ This hypothesis‐generating observational study points to the potentially deleterious role of the excessive inflammation on the outcome of severe pneumonia. Acute lung inflammation (ALI) caused by the Gram‐negative bacteria virulence factor LPS evokes the production of cytokines and chemokines in the bronchoalveolar space and increased trafficking of neutrophils that was suppressed by NTM, cSN50.1 peptide (Figure 4).^51^ As such, NTM potentially averts collateral damage to the fine structure of the air‐blood barrier in the lungs.

### 24.3 Microbial inflammation of the lungs due to MRSA

A microbial inflammation caused by community‐acquired methicillin‐resistant *Staphylococcus aureus* (MRSA) infections underlies a potentially fatal necrotizing pneumonia that complicates seasonal influenza outbreaks. Together with ventilator‐associated pneumonia caused by hospital‐acquired MRSA, these infections pose an increasing risk for ALI and its more severe stage, ARDS.^229^, ^230^ ALI caused by MRSA virulence factors, immunotoxins termed “superantigens,” was attenuated in an experimental model by NTM (cSN50 peptide) that both suppressed cytokine and chemokine production while reducing lung traffic of neutrophils, monocytes/macrophages and lymphocytes to the bronchoalveolar space. Moreover, NTM reduced lung microvascular injury manifested by increased permeability and protein leakage.^22^

### 24.4 Sepsis: microbial inflammation damaging microvessels

Sepsis represents the severe endothelial dysfunction in response to intravascular or extravascular infections causing reversible or irreversible injury to microcirculation responsible for multiple organ failure.^132^ Until now, over 100 trials in sepsis, including anti‐TNFα monoclonal antibody failed.^231^ In a clinically relevant, polymicrobial sepsis model, a combination of antimicrobial therapy with NTM increased survival to 55% as compared to 30% with antimicrobial therapy alone.^52^ Thus, NTM prevents a localized and systemic expression of mediators associated with a runaway microbial inflammation, while also enhancing the innate immunity–mediated clearance of bacteria that produced 700‐fold reduction of the bacterial burden on the lungs of septic animals prior to the onset of antimicrobial therapy.

### 24.5 Fulminant hepatitis

Fulminant hepatitis, a life‐threatening disease due to viral and non‐viral agents with increased incidence,^232^ is mediated by the inflammation‐driven apoptosis of the liver cells. Among an estimated 2 billion cases of viral hepatitis worldwide, approximately 20 million were projected to develop fulminant liver failure.^233^ Chronic hepatitis virus B and hepatitis virus C infections lead to the development of hepatocellular carcinoma, the third cancer‐related cause of death in developed countries.^234^ The loss‐of‐function mutations in *Sosc1* and *Socs3* genes, or their silencing during chronic inflammation, contribute to carcinogenesis of the liver and colon.^235^, ^236^ Intracellular protein therapy, with cell‐penetrating recombinant SOCS3 or SOCS1, may replenish the physiologically active SOCS in acute or chronic liver inflammation, as discussed above.

### 24.6 Microbial inflammation–driven apoptosis of the liver

**Figure 6:**
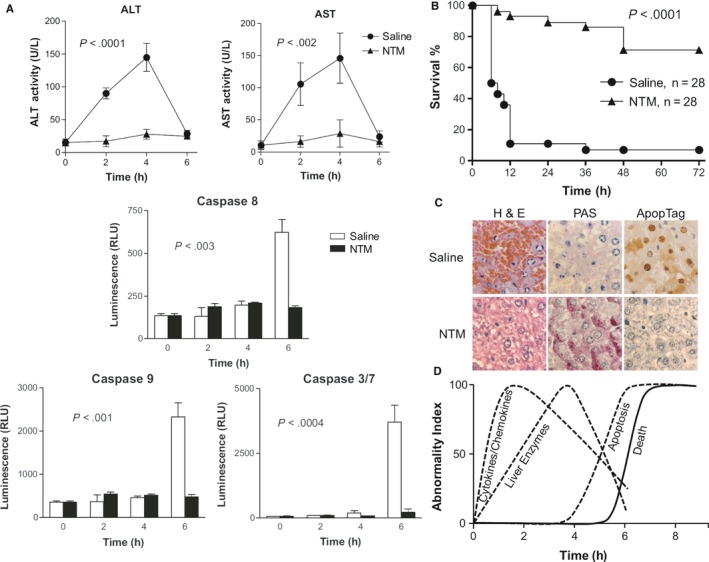
LPS‐induced liver injury in metabolically compromised mice is prevented by NTM treatment. A, Increased levels of liver enzymes (ALT and AST) in blood and liver caspases (8, 9 and 3/7) are significantly suppressed in animals treated with NTM. B, Survival in D‐Galactosamine‐sensitized mice challenged with LPS is significantly improved in animals treated with NTM. C, Liver injury depicted by haemorrhagic necrosis, glycogen depletion and apoptosis is attenuated in animals treated with NTM. D, Schematic depiction of time course of fulminant liver injury induced by LPS in metabolically compromised host mediated by cytokine/chemokine induction, hepatocyte injury marked by release of liver enzymes, apoptosis and death (adapted from Liu *et al*, J. Biol. Chem., 2004)

Experimentally, the inflammation‐driven liver cell apoptosis caused by microbial virulence factors occurs under the conditions of metabolic stress. In this context, the activation of liver macrophages (Kupffer cells) by LPS or stimulation of T lymphocytes by superantigen Staphylococcal Enterotoxin B induces massive liver apoptosis.^237^, ^238^ Persistent signalling to the nucleus through TLRs on Kupffer cells leads to the activation of genes that encode inflammatory mediators whose signalling cascades in hepatocytes contribute to their injury manifested by elevated liver enzymes in blood, followed by the apoptosis of metabolically compromised cells. A sequential analysis of these events indicates a lag phase of at least 4 hours before inflammation‐driven activation of caspases was detected in the liver (Figure 6A). Depending on the potency and duration of microbial insults, the physiologic suppressors of inflammation/apoptosis are overwhelmed. An intracellular protein or peptide therapy with cell‐penetrating recombinant SOCS3 or NTMs averts this massive liver injury^21^, ^237^, ^238^ (see Figures 3 and 6).

## 25 AUTOIMMUNE INFLAMMATION: TYPE 1 DIABETES

**Figure 7:**
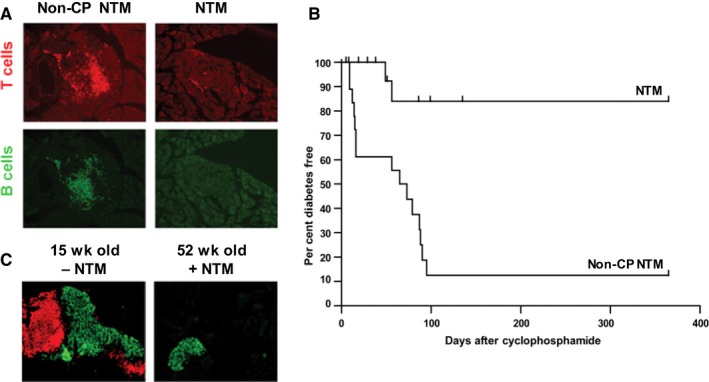
Autoimmune insulitis is attenuated by intracellular delivery of a nuclear transport modifier (MTM). A, Immunofluorescent analysis of pancreas obtained from cyclophosphamide (Cy)‐synchronized non‐obese diabetes (NOD) mice treated with NTM (cSN50 peptide) or non–cell‐penetrating NTM (cN50 peptide as inactive control). Pancreas section was stained with anti‐CD3‐PE (red, T cells) or anti‐B220‐FITC (green, B cells). B, Short‐term intracellular delivery of NTM, cSN50, protects NOD mice from autoimmune diabetes for over 1 y. C, 52‐week‐old Cy‐synchronized NOD mice show no signs of insulitis after short‐term treatment with NTM (right) as compared to the 15‐week‐old NOD mice from the beginning of experiment (adapted from Moore *et al*, PLoS ONE, 2010)

Type 1 diabetes (T1D) is a relentlessly devastating autoimmune disease that affects more than 10 million children, adolescents and adults worldwide, including an estimated 1.25 million Americans.^239^ The aberrant autoimmune reprogramming recruits T and B lymphocytes, macrophages and dendritic cells to the pancreatic islets and results in the gradual destruction of insulin‐producing beta cells by inflammation‐driven autophagy and apoptosis.^240^ The two‐stage process is proposed for the evolution of type 1 diabetes based on studying the microbiome in Finish children. In the first stage before the age of 5.5 years, the intestinal microbiome fails to programme the development of a competent immune system that protects against autoimmune diseases. Hence, the changes in the diversity of the gut microbiome set the stage for autoimmune diabetes. In the second step, the diversity of the microbiome declines towards the preponderance of *Bacteroides* sp over Firmicutes accompanied by the disappearance of *Bifidobacterium* sp and the lack of bacteria producing butyrate and lactate. At this point, the young child displays seroconversion towards diabetes‐associated autoantibodies.^241^ The innate immunity signalling pathway initiated by intestinal microbiota may drive pancreatic islet–based initiation of insulitis by macrophages.^242^ Ablation of the adaptor MyD88 in NOD mice not only rearranged the gut microbiota, but also prevented the development of autoimmune diabetes.^243^ As MyD88‐mediated signalling leads to the nuclear transport checkpoint (see Figure 2A), we hypothesized that the nuclear import of stress‐responsive transcription factors in innate and adaptive immune cells is required for the islet‐based autoimmune inflammation of Type 1 diabetes. With Daniel Moore, we tested this hypothesis in genetically prone NOD mice that resemble Type 1 “juvenile” diabetes in humans. A 2‐day course of intense NTM treatment administered to cyclophosphamide‐synchronized NOD mice resulted in a diabetes‐free state for 1 year without apparent toxicity or the need for insulin therapy.^244^ We found that NTM reversed the resistance of autoreactive T cells to activation‐induced cell death. The elimination of islet‐infiltrating, autoreactive B and T lymphocytes is a prominent feature of the NTM‐modulated process of beta cell protection (see Figure 7). Depletion of CD122‐positive CD8 T cells and NK cells with anti‐CD122 antibody in NOD mice protected them from ensuing autoimmune diabetes.^245^ Alternatively, imatinib, an inhibitor of tyrosine kinases, reduces ER stress in pancreatic β cells and reverses autoimmune diabetes.^246^ The islet‐protecting action of imatinib requires B lymphocytes.^247^

Individuals with T1D diabetes demonstrate a transition from autoimmune inflammation to atherosclerosis, a prime example of metabolic inflammation.^248^, ^249^ As mentioned earlier, we posed the question: Can NTM ameliorate metabolic inflammation?

## 26 METABOLIC INFLAMMATION

As we defined above, metabolic inflammation is the body's response to overfeeding and the excessive accumulation of metabolites (eg fatty acids, cholesteryl esters, triglycerides, amino acids, uric acid) due to inborn or acquired metabolic dysfunction observed in atherosclerosis, gout, homocystinuria, phenylketonuria and other disorders. This build‐up of metabolites alters homeostasis at the cell and organ levels. An elevated concentration of cholesteryl esters, triglycerides and other lipids in the blood, termed hyperlipidemia, represents a risk factor in cardiovascular and hepatobiliary morbidity and mortality worldwide.^250^ It places a huge burden on an estimated 35 million Americans with hypercholesterolaemia, with an annual death toll of over 500 000 due to cardiovascular disease underlying myocardial infarctions and strokes.^251^

### 26.1 Metabolic inflammation damaging macrovessels: atherosclerosis

Hyperlipidemia underlies atherosclerosis in lipid‐laden blood vessels.^103^ Hyperlipidemia due to elevated low‐density lipoproteins (LDL) cholesterol in blood combined with the low endothelial shear stress in these vessels promotes coronary plaque growth and vulnerability. This process is linked to the increased expression of genes regulated by the network of proinflammatory stress‐responsive transcription factors and metabolic transcription factors in immune and vascular cells.^252^, ^253^ The well‐established link between elevated blood LDL cholesterol and atherosclerosis was associated with an increased blood level of a general biomarker of inflammation, C‐reactive protein.^254^ This biomarker, as well as a rise of clotting factors such as fibrinogen, von Willebrand factor and complement proteins, reflects the acute phase protein response induced by the action of three mediators of inflammation, IL‐1β, IL‐6 and TNFα.^255^ Proinflammatory stress‐responsive transcription factors that control these mediators are widely distributed in vascular and immune cells, which accumulate in the dynamic process of atherosclerotic development.^256^, ^257^ Signalling cascades dependent on TLR2, TLR4 and MyD88 contribute to the development of atherosclerosis in apolipoprotein E‐deficient or LDL receptor‐deficient mice.^258^, ^259^ Importantly, NTM treatment in either the first 4 weeks or the last 4 weeks of the HFD protocol reduced atherosclerotic lesions by approximately 50% or approximately 30%, respectively, in LDL receptor–deficient mice.^116^ This reduction of preformed atherosclerotic plaque in the coronary sinus by NTM indicates that we have now ability to reverse experimental atherosclerosis in LDL receptor–deficient mice. Others reported a reduction of atherosclerosis in apolipoprotein E‐deficient mice fed a HFD and treated with NTM.^260^ Thus, NTMs are effective in atherosclerosis‐prone mice with LDL receptor or apolipoprotein E deficiency.

Consistent with the LDL receptor paradigm,^261^ statin mevinolin (lovastatin) and anti‐PCSK9 monoclonal antibodies do not lower cholesterol in familial hypercholesterolaemia homozygotes.^262^, ^263^ In contrast, the anti‐PCSK9 monoclonal antibody combined with statins reduced the risk of cardiovascular diseases in LDL receptor–sufficient individuals.^264^

### 26.2 Metabolic inflammation of the liver: from steatosis to steatohepatitis to end‐stage liver disease

Elevated levels of blood triglycerides contribute to the development of a fatty liver, termed steatosis, that evolves into non‐alcoholic steatohepatitis leading to liver cirrhosis. Four to twenty‐seven per cent of these individuals develop liver cancer, the predominant indication for liver transplantation in the United States.^265^ Consistent with the results of NTM action listed above in the animals fed a HFD, a fatty liver was averted, while elevated liver enzymes, ALT and AST, were normalized and the nuclear pool of phosphorylated NF‐κB Rel A was reduced as compared to controls^116^ (Figure 2D). Overnutrition with saturated fatty acid palmitate activates hepatocellular NF‐κB through BCL10, which is driven by the diacyl glycerol‐protein kinase C pathway.^266^ We posit that the activation of NF‐κB signalling pathway represents a “tipping point” in the transition of liver steatosis to steatohepatitis. NTM impedes the nuclear translocation of NF‐κB in HFD‐fed animals.^116^

Cumulatively, NTMs offer a new approach to the simultaneous reduction of atherosclerosis‐mediated coronary heart disease, steatohepatitis, hyperglycaemia and weight gain, the quartet of the growing menace of metabolic syndrome.

## 27 METABOLIC INFLAMMATION AND IMMUNOMETABOLISM

Immunometabolism has emerged as a research front focused on the metabolic pathways mostly in monocytes and macrophages.^267^ “Metabolic reprogramming” of these cells induced by microbial virulence factors such as LPS is controlled by changes in the mitochondria and transcriptional cascades. Cis‐aconitate decarboxylase (CAD), also known as the immune responsive gene 1 (IRG1), is induced by inflammatory stimuli such as LPS. CAD catalyses the production of itaconate.^268^ This reaction is linked to a succinate accumulation during innate immune response. As metabolic pathways, such as tricarboxylic acid metabolism, are mediated by enzymes and binding proteins, their expression level depends on the transcription factors akin to the synthetic pathways of cholesterol that is tightly regulated by the SREBPs‐dependent expression of the LDL receptor (see above). The product of IRG1, itaconate, inhibits succinate dehydrogenase.^269^ However, itaconate and its membrane‐penetrating derivative, dimethyl itaconate, induce the electrophilic stress that activates the transcription factors Nrf2‐ and ATF3‐mediated inhibition of IκBζ.^270^, ^271^ As transcriptional cascades mediated by Nrf2 (see Figure 2A) and ATF3 underlie the inflammatory response (see above), there is an apparent overlap between metabolic inflammation and immunometabolism.

## 28 ALLERGIC INFLAMMATION: ASTHMA

Asthma is mediated by allergic inflammation manifested by wheezing due to airway hyperresponsiveness and the obstruction of airflow. Over 100 genes that are linked to asthma susceptibility can modify its severity and response to treatment.^272^ Signalling to the nucleus in asthma is triggered by environmental insults (allergens, viruses, air pollutants, such as Diesel exhaust particles, chemicals, mites and moulds).^273^, ^274^ In response to these insults, allergic inflammation depends on the recruitment of ILC from the bone marrow^275^ and the activation of the CD4^+^ T lymphocytes (T_H_2) that produce potent inflammatory mediators, including type 2 cytokines, IL‐3, IL‐4, IL‐5 and IL‐13.^276^ The activation of T_H_2 CD4^+^ occurs through IL‐4 and IL‐13 receptors leading to the phosphorylation of STAT6 by JAKs and engagement of two other transcription factors, transacting T cell–specific transcription factor GATA3 and c‐Maf. They target a set of genes located on chromosome 5 that transcribe the mediators of allergic inflammation.^276^ Moreover, in lung epithelial cells, the airway allergic signalling is initiated upon the stimulation of the protease‐activated receptor‐2 (PAR2) and the G protein pathway that leads to the transcription factor NF‐κB1‐mediated expression of IL‐4. Whereas NF‐κB1, STAT6 and c‐Maf predominantly regulate gene encoding IL‐4,^277^ GATA3 modulates genes that encode the mediators of allergic inflammation, type 2 cytokines (IL‐3, IL‐4, IL‐5 and IL‐13). The nuclear transport of these transcription factors awaits analysis. In the meantime, the first anti‐IgE–humanized monoclonal antibody was approved for the treatment of allergic asthma.^278^

### 28.1 Asthma and metabolic inflammation

Metabolic inflammation contributes to the increased asthma incidence in obesity. Hence, systemic glucocorticosteroids are contraindicated due to their known side effects including diabetes, weight gain and osteoporosis.^279^ Along with obesity, Type 2 diabetes also predisposes to bronchial hyperresponsiveness. The glucagon‐like peptide (GLP) 1 increases glucose‐stimulated insulin secretion while reducing glucagon release. Its receptor, GLP1‐R, is displayed in human lung.^280^ In human isolated airways, the GLP‐1R agonist, extendin‐4, prevented bronchial hyperresponsiveness in the presence of high glucose. Moreover, GLP‐1R‐evoked signalling suppresses IL‐33 production, thereby preventing the ILC2 response to protease‐containing aeroallergens.^281^

Cumulatively, allergic inflammation superimposed on metabolic inflammation requires a dual anti‐inflammatory strategy while avoiding metabolically harmful glucocorticoids.

## 29 PHYSICAL (POST‐TRAUMATIC) INFLAMMATION DEPENDS ON NUCLEAR TRANSPORT OF TRANSCRIPTION FACTORS

Injury is one of the most important public health problems in the United States. It is responsible for more lost years of productive life than cancer and heart disease combined.^282^ Critically injured patients suffering from trauma and burns displayed, in their peripheral blood leucocytes, a broad spectrum of activated genes that encode inflammatory cytokines and chemokines, signal transducers (COX 2 and nitric oxide synthase) and cell adhesion molecules. This response to trauma and burns, dubbed the “genomic storm,” represents inflammation caused by physical insults. A similar response was found in human volunteers who were challenged with LPS as an inducer of microbial inflammation^49^ (see above). The similarity of both responses indicates a common mechanism of the genomic storm that is mediated by stress‐responsive transcription factors and can be calmed by NTM.^51^

It is thus not surprising that NTM (SN50 peptide) was effective in an experimental model of traumatic brain injury.^283^ The N‐methyl‐D‐aspartate glutamate receptors (NMDAR) play a role in neuronal death after brain trauma and stroke. NMDAR activates the SREBP1 transcriptional pathway.^284^ The SREBP1 pathway inhibitor targeting insulin‐induced gene‐1 (Insig‐1) prevented SREBP1 activation and attendant neuronal damage. It is likely that the beneficial effect of NTM on traumatic brain injury^283^ can also be attributed to its targeting of importin β1, thereby suppressing expression of SREBP1, as well as controlling four stress‐responsive transcription factors cascades (see Figure 2A,C).

## 30 THE FIVE SIGNS OF INFLAMMATION: 2000 YEARS LATER

Celsus’ five cardinal signs of inflammation have their modern mechanistic underpinnings. Redness and swelling (see Figure 1) represent the vasodilation and increased vascular permeability responsible for microvascular leaks. Brain and lung oedema are the most severe forms of microvascular leaks.^285^ Small blood vessels (<100 μm in diameter) comprising the microcirculation of multiple organs are primarily affected by microbial, autoimmune and allergic inflammation. The end stage of microbial inflammation, sepsis and septic shock, represents severe endothelial dysfunction, causing the reversible or irreversible injury to microcirculation which is responsible for multiple organ failure.^132^

Pain is evoked by injury registered by sensory axons. They emit signals to the neuronal cell body conveyed to the nucleus by the microtubule motor dynein and nuclear transport shuttles.^286^ One of them, importin α5, is colocalized with transcription factor STAT 3 and dynein.^287^ Pain is also mediated by acid‐sensing ion channels.^288^

Fever is induced by exogenous and/or endogenous substances termed “pyrogens,” such as the virulence factor of Gram‐negative bacteria, LPS, also known as endotoxin, and cytokines, such as interleukin (IL)‐1β.^289^, ^290^ The pyrogens are sensed by their cognate receptors on brain microvascular endothelial cells that form the neurovascular unit proximal to the brain thermoregulatory centre.^291^ Therein, pyrogens trigger a signalling cascade mediated by the TGFβ‐activated kinase 1 (TAK1), a MAP3 kinase, that regulates the expression of cyclooxygenase 2 (COX2) and the subsequent production of prostaglandin E2^140^ suppressed by aspirin and other non‐steroidal anti‐inflammatory drugs (NSAIDs).

## 31 THE PREVENTION OF DISEASES MEDIATED BY INFLAMMATION

The causative approach to the diseases mediated by microbial, autoimmune, allergic, metabolic and physical inflammation (see Table 1) holds the key to their prevention. Immunoprophylaxis based on vaccination is the most effective and economically sound approach to diseases mediated by microbial inflammation.^292^ Its lessons are also being applied to the prevention of metabolic inflammation that mediates atherosclerosis and its complications, such as heart attacks and strokes (see below). Diet, exercise and drugs lower the risk of these diseases.^103^ However, the effectiveness of prevention depends on behavioural and home economic factors as well as better methods of early detection and precise monitoring. Fortunately, alternative immunoprophylactic approaches are under development.

Two vaccines, based on the peptides representing the human apolipoprotein B100, a constituent of LDL, showed specific binding to human HLA haplotypes. Immunization of humanized ApoB100‐transgenic mice that are LDL receptor‐deficient, a model for human familial hypercholesterolaemia, produced encouraging results of markedly reduced macrophage infiltration and diminished size of atherosclerotic plaques in vaccinated mice.^293^ Altogether, these advances bode well for the immunoprophylaxis of diseases mediated by microbial and metabolic inflammation in millions of people worldwide.

Preventing injuries that cause diseases mediated by physical inflammation was codified in the United States by the Injury Prevention Act of 1986. It was followed by significant progress in preventing unintentional injury, a reduction in violence‐related trauma and an improvement in trauma care.^282^

## 32 SUMMARY AND CONCLUSIONS

Most human diseases are mediated by inflammation caused by microbial, autoimmune, allergic, metabolic, physical and constitutive factors. The focus of inflammation research has shifted from the phagocytes, discovered by Mechnikov more than a century ago, to the cell's nuclear landscape moulded by the inflammatory regulatory networks of the genome. The present‐day “inflammatory regulome” is continually explored and refined, providing the much‐needed basis for a better understanding of the mechanism of dysregulated control of the inflammatory response.

We present an integrated, cause‐oriented view of inflammation that offers a new approach to its regulation. Control of the accessibility of transcription factors to gene regulatory networks at the nuclear port of entry, and elsewhere, reduces the expression of multiple genes that encode the mediators of inflammation. This nucleocentric strategy for inflammation applies to innate and adaptive immunity that is interwoven with inflammation caused by multiple insults. Rapid recognition of these insults and their elimination by immunoprophylaxis offers the most effective aetiologic approach to microbial and possibly other types of inflammation. Preventive vaccines that limit the entry and spread of microbial pathogens could tame both runaway microbial inflammation and collateral organ injury caused by microbial agents increasingly resistant to antimicrobial therapy. Control of the inflammatory regulome by a new generation of therapeutics that extinguish the genomic storm in most life‐threatening inflammatory states (eg septic shock, major trauma) and chronic genomic reprogramming are within reach of practicality.

Restraining and eliminating the clones of autoreactive immune cells by reprogramming their genome is also plausible in autoimmune inflammation that damages multiple organs, the causative mechanism of Type 1 diabetes, rheumatoid arthritis, lupus, multiple sclerosis, psoriasis and other similar diseases. Reciprocal interaction of innate and adaptive immunity with gut microbiota that comprise 10^13^ organisms with diverse genomes offers untapped potential for the prevention and more effective control of autoimmune inflammation that underlie Type 1 diabetes and Crohn's disease.

New approaches applied to metabolic syndrome mediated by inflammation are especially needed since behavioural modifications such as a healthy diet and exercise, albeit effective, are frustratingly unpopular in developed countries. Therefore, we anticipate a shift from targeting the single mediators of metabolic inflammation to new, more comprehensive measures that will restore the human genome to a physiologically balanced state.

Bioengineered, cell‐penetrating recombinant SOCS3 comprises a new class of anti‐inflammatory intracellular therapy for acute liver inflammation and possibly inflammatory injury of other organs. Cell‐penetrating NTMs provide a wide‐ranging, experimental therapy of microbial, autoimmune, metabolic and physical (post‐traumatic) inflammation. These evolving intracellular therapies dismantle the inflammatory regulome instead of inactivating a single gene product that encodes an inflammatory mediator. These new therapies complement current extracellular therapies that individually inactivate either the multiple mediators of inflammation or their cognate receptors.

Acute and chronic diseases mediated by inflammation are increasingly aggravated by the excessive use of opiates for inflammatory pain, long‐lasting disability and cognitive decline of their victims. Reducing the extent and duration of inflammation caused by microbial, autoimmune, constitutive, allergic, metabolic and physical factors will beneficially effect the quality of life, economy and longevity of the global population.

## CONFLICT OF INTEREST

Jacek Hawiger and Jozef Zienkiewicz are co‐inventors of multiple patents issued and pending relating to cell‐penetrating peptides and proteins and their use for anti‐inflammatory therapy. All rights are assigned to Vanderbilt University.

## AUTHOR CONTRIBUTIONS

JH conceived and outlined this review manuscript, wrote the paper and approved the final version of the manuscript. JZ assisted in writing and editing this review manuscript, collected the data and prepared figures.
